# Alteration of Proteotranscriptomic Landscape Reveals the Transcriptional Regulatory Circuits Controlling Key-Signaling Pathways and Metabolic Reprogramming During Tumor Evolution

**DOI:** 10.3389/fcell.2020.586479

**Published:** 2020-12-15

**Authors:** Geoffroy Andrieux, Sajib Chakraborty, Tonmoy Das, Melanie Boerries

**Affiliations:** ^1^Faculty of Medicine, Medical Center–University of Freiburg, Institute of Medical Bioinformatics and Systems Medicine, University of Freiburg, Freiburg, Germany; ^2^German Cancer Consortium (DKTK) Partner Site Freiburg, German Cancer Research Center (DKFZ), Heidelberg, Germany; ^3^Molecular Systems Biology Laboratory, Department of Biochemistry and Molecular Biology, University of Dhaka, Dhaka, Bangladesh; ^4^Comprehensive Cancer Center Freiburg, Medical Center–University of Freiburg, University of Freiburg, Freiburg, Germany

**Keywords:** proteomics, multi-omics, cancer, transcriptional network, mRNA-to-protein correlation, mRNA-sequencing

## Abstract

The proteotranscriptomic landscape depends on the transcription, mRNA-turnover, translation, and regulated-destruction of proteins. Gene-specific mRNA-to-protein correlation is the consequence of the dynamic interplays of the different regulatory processes of proteotranscriptomic landscape. So far, the critical impact of mRNA and protein stability on their subsequent correlation on a global scale remained unresolved. Whether the mRNA-to-protein correlations are constrained by their stability and conserved across mammalian species including human is unknown. Moreover, whether the stability-dependent correlation pattern is altered in the tumor has not been explored. To establish the quantitative relationship between stability and correlation between mRNA and protein levels, we performed a multi-omics data integration study across mammalian systems including diverse types of human tissues and cell lines in a genome-wide manner. The current study illuminated an important aspect of the mammalian proteotranscriptomic landscape by providing evidence that stability-constrained mRNA-to-protein correlation follows a hierarchical pattern that remains conserved across different tissues and mammalian species. By analyzing the tumor and non-tumor tissues, we further illustrated that mRNA-to-protein correlations deviate in tumor tissues. By gene-centric analysis, we harnessed the hierarchical correlation patterns to identify altered mRNA-to-protein correlation in tumors and characterized the tumor correlation-enhancing and -repressing genes. We elucidated the transcriptional regulatory circuits controlling the correlation-enhancing and -repressing genes that are associated with metabolic reprogramming and cancer-associated pathways in tumor tissue. By tightly controlling the mRNA-to-protein correlation of specific genes, the transcriptional regulatory circuits may enable the tumor cells to evolve in varying tumor microenvironment. The mRNA-to-protein correlation analysis thus can serve as a unique approach to identify the pathways prioritized by the tumor cells at different clinical stages. The component of transcriptional regulatory circuits identified by the current study can serve as potential candidates for stage-dependent anticancer therapy.

## Introduction

Production and maintenance of cellular transcriptome and proteome demand a series of interconnected processes ranging from mRNA production, processing, and degradation to protein production, modification, and regulated-destruction (Vogel and Marcotte, 2012). The dynamic balance among these processes ultimately determines the mRNA and protein abundances for a given gene. Previously, several large-scale multi-omics studies involving transcriptomics and proteomics in a diverse range of cells and tissues of mouse and human origin have shown that mRNAs and their corresponding proteins levels are moderately correlated (coefficient of correlation *R* ≤ 0.4) (Schwanhausser et al., 2011; Kristensen et al., 2013; Wilhelm et al., 2014). This moderate mRNA-to-protein correlation roughly indicates that <40% variation on the protein levels is ascribed to the mRNA levels, while the remaining variance (>60%) is contributed by the combined manifestation of differences in protein production and degradation rates. The extent of contributions from the different processes in the abundance variation of proteome has been identified in proliferating (Schwanhausser et al., 2011), differentiating (Kristensen et al., 2013), and stimulated (Jovanovic et al., 2015) murine immune cells, showing a varying degree of contribution of transcription, translation, and protein degradation. For instance, Schwanhausser et al. (2011) demonstrated that the transcription attributes to 34%, whereas translation explains 55% of variation on the protein levels in proliferating NIH3T3 mouse fibroblast cell line. On the other hand, Kristensen et al. (2013) utilizing differentiating human THP-1 myelomonocytic leukemia cell line showed that protein abundance variance can be explained efficiently by considering both the protein production and degradation rates contributing to 41 and 13% of proteome variation. Interestingly, the notion of translational process as the dominant factor to determine the protein abundance over other processes has been challenged by Jovanovic et al., and indeed, by estimating the transcription, translation, and protein degradation before and after lipopolysaccharide (LPS) stimulation in mouse bone marrow–derived dendritic cells (BMDCs), the authors identified that before stimulation mRNA levels contribute more than protein translation and degradation rates to overall protein abundance (Jovanovic et al., 2015). The stimulation of LPS produced a dramatic result in BMDCs where the changes in mRNA levels were shown to play an even more dominant role to control protein variation (Jovanovic et al., 2015). Apart from the mRNA levels, transcriptome-wide RNA structure characterization suggested that mRNA stability owing to the secondary structure may play an important role in regulating protein abundance on a global scale (Ding et al., 2014; Wan et al., 2014). Altogether these results suggested transcription and translational processes in concert with the respective stability of mRNAs, and proteins control the proteotranscriptomic landscape at a given time in mammalian cells. mRNA-to-protein correlation stemming from their respective abundances is the end product of the dynamic interplay among mRNA/protein production and degradation processes.

The impact of mRNA and corresponding protein stability on their respective abundance and hence their correlation has not been investigated systematically in a genome-wide manner. Lack of studies devoted to genome-wide measurements of human mRNA and protein half-lives poses a challenge to investigate the relationship of the stability of mRNA and protein to their correlation. A breakthrough came when Schwanhausser et al. quantified the half-lives of thousands of mRNA and their corresponding proteins in murine cells and classified the entire genome into four categories based on the stability profile of mRNA and proteins—stable mRNA–stable protein, unstable mRNA–stable protein, stable mRNA–unstable protein, and unstable mRNA–unstable protein. This stability profiling of mRNAs and corresponding proteins in mammalian cells presents an opportunity to address fundamental questions about the relationship between mRNA/protein half-lives and their correlation pattern. For instance, the impact of mRNA and protein stability on their subsequent correlation on a global scale has not been investigated. Moreover, whether the stability-dependent correlation pattern is conserved across human tissues and cell lines or has a tissue-specific pattern remains unresolved. More importantly, whether this stability-dependent correlation pattern is modified in altered cellular states, such as cancer has not been fully established. Finally, identification of the gene-specific altered mRNA-to-protein correlation in cancer state may enable us to dissect the relevant pathways that are under transcriptional controls and lead to the identification of deregulated transcriptional circuits in cancer. Kosti et al. (2016) identified that mRNA-to-protein correlation is higher in normal tissue compared to their cancer counterparts for only 153 genes across 10 different cancer types where the authors have used reversed-phase protein array (RPPA) dataset from the Cancer Proteome Atlas. Unlike LC-MS/MS-based proteomics, the RPPA—an immunoassay-based technique (Spurrier et al., 2008)—can measure only a few hundred proteins because of the availability of antibodies and may be subjected to non-specific binding of antibodies resulting in lower reproducibility of results (Chakraborty et al., 2018). Whether this lower mRNA-to-protein correlation of a few hundred genes has biological significance and also applicable for genome-wide scale is highly questionable. Additionally, the study did not address the influence of mRNA and protein stability on the differential correlation between normal and cancer tissues. On the contrary, Tang et al. (2018) identified a global higher mRNA-to-protein correlation in breast tumors (*n* = 65) compared to adjacent non-cancerous tissues (*n* = 53) and subsequently showed that the highly correlated genes were enriched in protein processing and disease metabolic pathways. Interestingly, the authors utilized predicted mRNA and protein stability profiles and showed that the global mRNA-to-protein correlation increased with the predicted stability of corresponding mRNA and protein molecules (Tang et al., 2018). Whether the global trend of higher mRNA-to-protein correlation in tumor tissue is restricted only to breast cancer or conserved across different cancer types has not been investigated.

The genome-wide stability profile of mRNAs and proteins provides a unique opportunity to interrogate the relation of the mRNA and protein half-lives with their respective correlation. To address these questions, we took the advantage of stability profiling as described by Schwanhausser et al. and investigated their mRNA-to-protein correlation pattern across human tissues and cell lines. With the aim to draw the association between stability and correlation between transcriptome and proteome, here we present a multi-omics data integration study of mRNA and protein correlation across murine and human tissues in different stability groups in a genome-wide manner. In order to gain insight into altered cellular conditions in cancer state, we compared the stability group–specific transcriptome vs. proteome correlation of normal tissues, cell lines, colon cancer tissues, and non-tumor colon tissues. Finally, we extended out our analysis to illustrate the association between molecular features of colon cancer patients and the variation of the transcriptome to proteome correlations. Altogether by developing a robust multi-omics integration strategy, we showed the utility of mRNA-to-protein correlation analysis to identify molecular features across mammalian systems.

## Methods and Materials

### Datasets Description

**Figure 1:**
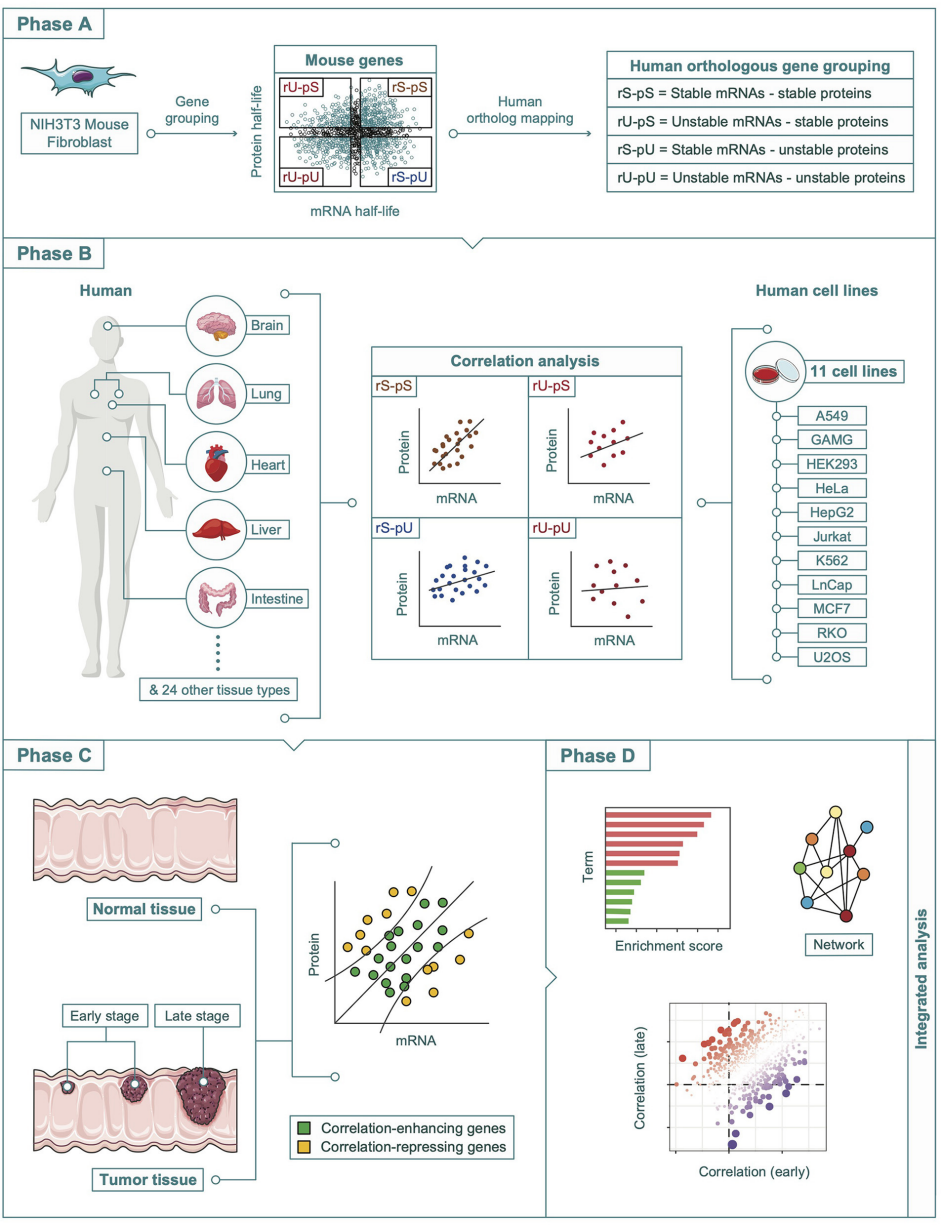
A schematic diagram showing the multi-omics integration pipeline. The analysis pipeline was divided into four phases. Phase A: Determination of the mouse-derived stability-dependent mRNA-to-protein correlation patterns followed by the mapping of human orthologous genes. Phase B: Validation of the stability-dependent mRNA-to-protein correlation patterns in human tissues, and human cell lines. Phase C: Investigation of the alteration in correlation pattern in tumor and non-tumor tissues. Phase D: Integrative multi-omics analyses including enrichment, network, and correlation analyses.

All the datasets that were analyzed in this study were retrieved from publicly available resources. A complete description of all the datasets herein is provided as Supplementary Table 1. A schematic diagram representing an overview of the overall study design, data sources, and methods used in this article is shown in Figure 1.

#### Classification of the Genes Based on the mRNA and Protein Stability

mRNA and proteins half-lives data generated by metabolic pulse labeling experiments using NIH3T3 mouse fibroblasts cells were retrieved from Schwanhausser et al. (2011) (Bioproject: PRJNA80021) (Supplementary Table 2). As defined by Schwanhausser et al., mRNAs and proteins were considered stable if the half-lives were higher than 10 and 75 h, respectively; or unstable if the half-lives were lower than 8 and 50 h, respectively. Murine genes were therefore divided into four groups based on mRNA and protein stability: mRNA-stable, protein-stable (rS-pS, *n* = 1,729); mRNA-unstable, protein-stable (rU-pS, *n* = 594); mRNA-stable, protein-unstable (rS-pU, *n* = 2,110); and mRNA-unstable, protein-unstable (rU-pU, *n* = 1,836). A fifth group containing all the genes with mRNA half-life between 8 and 10 h or protein half-life between 50 and 75 h was considered as “unclassified” (*n* = 3,044). The mouse genes were subsequently mapped to orthologous human genes where genes with missing human orthologs were excluded. The genes that were not classified in a particular stability group were considered as “unclassified” human orthologs. The matching of mouse–human ortholog resulted in the final human stability groups: rS-pS (*n* = 830 genes), rU-pS (*n* = 311 genes), rS-pU (*n* = 1,001 genes), rU-pU (*n* = 899 genes), and unclassified (*n* = 1,475 genes) groups (Supplementary Table 2).

#### Analysis of the Ribosomal Footprinting Densities in Mouse and Human Cells

Ribosomal footprinting (RFP) densities of 6,250 genes from mouse BMDCs were retrieved from Jovanovic et al. (2015) (Bioproject: PRJNA256211). The dataset consisting of murine gene-specific RFP density values in biological duplicates was matched with the stability profiling dataset as described above. A total of 2,321 matched genes were identified for which both the RFP density and stability profiling were available. For human cells, RFP densities of 11,000 mRNA transcripts were obtained from human HEK293 cells from Calviello et al. (2016) (Bioproject: PRJNA296059). The RFP datasets of HEK-293 cells were mapped to orthologous mouse genes with stability profiles. Finally, the intersection genes (*n* = 1,950 genes) between mouse BMDCs and human HEK-293 cells have been used for analysis (Supplementary Table 3).

#### Transcriptome and Proteome Dataset Retrieval From Human Tissues and Cell Lines

For human tissues, we took advantage of the different available datasets representing mRNA and protein abundances in a genome-wide manner to define three independent datasets. First, we retrieved proteomics data from Kim et al. (2014), where they performed proteome profiling of 17,294 genes from 17 adult tissues as part of the human proteome map (HPM). The mRNA abundance data was retrieved from The Genotype-Tissue Expression (GTEx) (Consortium, 2013). In the current article, the mRNA expression values representing the mean across several samples from GTEx were used. We followed the strategy previously described by Kosti et al. (2016) to combine the GTEx and HPM datasets. Briefly, the combination of GTEx and HPM datasets resulted in the 14 overlapping tissues (adrenal gland, bladder, colon, esophagus, frontal cortex, heart, kidney, liver, lung, ovary, pancreas, prostate, spinal cord, and testis). The combined mRNA (GTEx) and protein (HPM datasets) abundances of the matched genes were obtained and mapped against the stability groups. A total of 4,186 genes with mRNA and protein abundance were identified for which stability profiling was available. This combination of GTEx and Kim et al. datasets was defined as Dataset 1 (Supplementary Table 4). Second, retrieval of mRNA and protein abundances from 12 different healthy human tissues (uterus, kidney, testis, pancreas, stomach, prostate, ovary, thyroid gland, adrenal gland, salivary gland, spleen, and esophagus) as described by Wilhelm et al. (2014) and subsequent mapping to stability groups yielded 3,295 genes (Dataset 2) (Supplementary Table 4). Lastly, we obtained mRNA and protein abundance data from Wang et al., where the authors performed deep-transcriptome and -proteome profiling of 29 paired human tissues (tonsil, liver, spleen, stomach, brain, lung, testis, duodenum, small intestine, urinary bladder, gall bladder, esophagus, heart, thyroid, endometrium, colon, fallopian tube, kidney, smooth muscle, prostate, appendix, pancreas, ovary, placenta, rectum, fat, lymph node, salivary gland, and adrenal gland) (Wang et al., 2019). A total of 4,328 number of genes was obtained after the mapping of the genes to stability profiling groups (Dataset 3) (Supplementary Table 4).

To obtain transcriptome and proteome from cell line, we retrieved the mRNA and protein abundances from 11 cell lines (A549, GAMG, HELA, HEK293, HEPG2, JURKAT, K562, LNCAP, MCF7, RKO, U2OS) as described by Geiger et al. (2012) and compared with the stability groups to stratify 4,390 genes with matched stability profiling data (Supplementary Table 5).

#### Transcriptome and Proteome Dataset Retrieval From Human Tumor and Non-tumor Tissues

The mRNA and protein abundance datasets from 58 tumor tissues were obtained from The Cancer Genome Atlas (TCGA) (Tomczak et al., 2015) and Clinical Proteomic Tumor Analysis Consortium (CPTAC) (Edwards et al., 2015), respectively (Zhang et al., 2014) using the TCGAbiolinks R package (Mounir et al., 2019). In addition, mRNA abundance data from 41 donor-matched non-tumor colon tissues were retrieved from TCGA. Finally, proteomics data from 30 non-tumor colon tissues were retrieved from the CPTAC database. The clinicopathological data including histologic subtype and tumor location as well as demographic features (gender and age) of the donors of the 58 colon tumor samples are given in Supplementary Table 1. From these datasets, 2,357 genes with quantified mRNA and proteins were mapped to the stability groups (Supplementary Table 6). Additionally, we retrieved the tumor purity estimates for colon adenocarcinoma (COAD) samples from Aran et al. (2015), where the authors used different measurement modalities of tumor purity. Briefly, we retrieved immunohistochemistry (IHC) (as estimated by image analysis of hematoxylin and eosin–stained slides) and consensus purity estimate (CPE) scores (Aran et al., 2015). COAD patients were divided into three groups (high, intermediate, and low, as described above) based on the tumor purity scores using three-quantiles. The mRNA-to-protein correlations were then compared among the different purity groups.

The web resources of the data repositories of the transcriptomic and proteomic datasets are given in Supplementary Table 1 and the Web Resources section.

### Correlation and Bootstrap Analysis

The datasets from healthy human tissue and cell line samples were made uniform by keeping only the prestratified genes based on stability groups (rS-pS, rU-pS, rS-pU, and rU-pU) that were quantified in all samples. The mRNA-to-protein Spearman rank correlation was calculated for each stability group individually. To assess the significance of a given stability group compared to a random set of genes, a bootstrap analysis was performed. Briefly, for a given stability group with “n” genes, the same number of genes were randomly selected 1,000 times among the whole set of genes followed by the comparison of the observed Spearman correlation value to the 1,000 correlation values obtained with random genes. Upper- and lower-tail *p*-values were calculated from the empirical cumulative distribution to determine whether the observed correlations were higher or lower than expected by chance. False Discovery Rate (FDR) below 0.05 was considered as significant.

The tissue and cell lines were matched according to the cell-type origin as described by Chakraborty et al. (2019). For instance, the A549 cell line of lung epithelial origin was matched with lung tissue. Similarly, GAMG, HEPG2, LNCAP, and RKO cell lines were matched to the frontal cortex, liver, prostate, and colon tissues, respectively. For each tissue–cell line pair, we calculated the mRNA–protein correlation (Spearman rank correlation) in all four stability groups. To assess the significance of the difference of mRNA-to-protein correlation between tissues and cell lines, we performed a similar bootstrap analysis as described above by selecting 1,000 random sets of genes per stability group and compared the observed difference of correlation to the 1,000 random ones. Finally, we calculated upper- and lower-tail *p*-values from empirical cumulative distribution.

### Unpaired Analysis of mRNA-to-Protein Correlation Between Tumor and Non-tumor Samples

For comparing tumor and non-tumor tissues, a paired analysis could not be performed because of the lack of patient-matched paired protein data for non-tumor colon tissue. To overcome this, we conducted an unpaired analysis by randomly selecting 20 independent, i.e., non-paired, samples for each of the categories: non-tumor tissue RNA, tumor–tissue RNA, non-tumor tissue protein, and tumor–tissue protein. Altogether, these 80 samples represented different patients to ensure the independence of the samples and thus were suitable for unpaired analysis. The mRNA-to-protein Spearman correlation was calculated for all possible combinations from non-tumor and tumor samples (400 combinations each). A schematic diagram illustrating the randomized unpaired analysis pipeline is given (Supplementary Figure 1).

#### Patient-Wise Correlation Analysis Between Tumor and Non-tumor Tissues

The overall difference of mRNA-to-protein correlation between tumor and non-tumor tissues was determined by a non-parametric two-sided Wilcoxon test. To assess the significance of the difference of correlation between tumor and non-tumor tissues in each stability group, we performed a similar bootstrap analysis as described above. Briefly, for each stability group with “n” genes, the same number of genes were randomly selected 1,000 times from the whole set of genes. The lower- and upper-tail probability of the median observed differences between non-tumor and tumor was estimated from the normal distribution of the 1,000 median differences from the random sets of genes in each stability group.

#### Gene-Centric Analysis of mRNA-to-Protein Correlation

For each gene, we calculated the ranks between mRNA and protein to determine the influence of a gene on the mRNA-to-protein correlation value. Genes with a small difference of rank tend to increase the absolute correlation value, whereas genes with a high difference of rank tend to decrease the absolute correlation value. Hence, by comparing the difference of ranks in tumor and non-tumor tissues, we can identify genes that are contributing to the increased (correlation-enhancing genes) or decreased (correlation-repressing genes) absolute correlation in tumors. For each gene, a one-sided Wilcoxon rank test was used to get the upper- and lower-tail *p*-values of the difference between tumor and non-tumor tissues.

### mRNA-to-Protein Correlation Analysis in the Early and Late Stage of Colon Cancer

TCGA colon tumor (COAD) samples (*n* = 58) were divided into early stage (*n* = 32), representing stages I and II patients, and late stage (*n* = 26), representing stages III and IV patients (Supplementary Table 7). Patient-matched mRNA and protein abundances from 58 COAD samples allowed a paired analysis of mRNA-to-protein correlation in the early- and late-stage groups separately. The difference between early- and late-stage mRNA-to-protein correlation was evaluated by the same bootstrap analysis as described above.

### Transcription Factor–Target Gene Network Analysis

The transcription factor (TF)–target genes dataset generated by TF-binding site profiles as measured by the ChIP-seq technique were retrieved from the ENCODE database (Consortium, 2011). The association between a target gene and corresponding TF was determined by the binding probability of a TF near the transcription start site of a gene. The TF–target gene dataset was then mapped into the gene list for which a significant deviation of the correlation was observed in tumors compared to non-tumor samples. The mode of function of the TFs was obtained from the UniPort database (The UniProt, 2017) and TRRUST (Han et al., 2018). The TF–target gene dataset was mapped into the gene list for which a significant deviation of the correlation was observed in tumors compared to non-tumor samples. The networks have been constructed based on a manually curated TF–target gene network among the correlation-enhancing and correlation-repressing genes. The network representing the TF–target genes associations was visualized by using Cytoscape (Shannon et al., 2003).

### Statistical Analysis for Dataset Comparison

When comparing two datasets, we investigated whether the data followed a normal distribution using a Shapiro test (FDR <0.05 was used as a significant threshold). Therefore, the significance of the difference between the two data distributions was estimated using a two-sided *t*-test in case of a normal distribution or using a two-sided Wilcoxon test otherwise. In both cases, FDR <0.05 was considered as significant. Results of the Shapiro test regarding the data-distribution patterns are given in Supplementary Table 8.

### Gene-Set Enrichment Analysis

Fisher exact was used to determine whether a gene set was enriched or not in tumor correlation-enhancing or -repressing genes. Within each stability group, genes were ranked according to the absolute delta rank comparing non-tumor against tumor condition. The enriched gene sets from the top 25% extreme values (i.e., 166, 40, 127, and 50 in rS-pS, rU-pS, rS-pU, and rU-pU, respectively) were identified using the ConsensuspathDB (Kamburov et al., 2009). The whole gene set in each stability group was used as background in the enrichment analysis. Moreover, we analyzed the molecular features of COAD. To this end, we performed a single-sample gene-set enrichment using Hallmark gene sets from MSigDB (Subramanian et al., 2005). We first normalized each COAD sample by subtracting the average mRNA expression value measured in normal colon tissue from TCGA. Then a Normalized Enrichment Score (NES) was calculated for each hallmark term in each tumor sample using the fgsea R package (Sergushichev, 2016). In the next step, the patients were classified into three different groups (high, intermediate, and low NES) based on the three-quantiles for each of the molecular features. Subsequently, for each hallmark, the mRNA-to-protein correlation was compared among the three different groups to identify any significant deviation.

## Results

### The Different Stability Groups Influence the Overall mRNA and Protein Abundance in Mouse Cells

To understand the impact of mRNA and corresponding protein half-lives on their abundances, we took advantage of the four stability groups (rS-pS, rU-pU, rS-pU, and rU-pU) in mouse fibroblast cells as described by Schwanhausser et al. (Figure 1 and Supplementary Table 2) and compared the mRNA and protein abundance of different stability groups (Supplementary Figure 2). Results showed the genes with stable mRNAs irrespectively of protein stability (rS-pS and rS-pU) showed significantly higher mRNA abundance compared to those with unstable mRNAs (rU-pS and rU-pU) (two-sided Wilcoxon test FDR = 0). Similarly, genes with stable proteins with or without stable mRNAs (rS-pS and rU-pS) showed a significantly higher abundance of proteins than the genes with unstable proteins (two-sided Wilcoxon test FDR = 0). This result indicates that mRNA/protein stability is likely to play an important role in determining their abundances. The significant impact of stability on murine mRNA and corresponding protein abundances raises the possibility that stability may also affect the mRNA and protein abundance and hence their correlation in a different species, such as human. A schematic diagram in Figure 1 shows how the stability-dependent mRNA-to-protein correlation has been analyzed in the mouse, as well as human cells and tissues.

### Ribosomal Footprinting Density Map Correlates mRNA/Protein Stability Profiles in Murine and Human Cells in a Gene-Specific Manner

We asked whether the impact of mRNA and protein stability is conserved across mammalian species including humans. To this end, we took advantage of RFP density maps that represent the localization of active ribosomes over the entire length of mRNA transcripts in both mouse BMDCs (Jovanovic et al., 2015) and human HEK-293 cells (Calviello et al., 2016). We investigated whether the genes with differential mRNA/protein stability have a varying degree of RFP densities and importantly if the RFP densities in different stability groups are similar between mouse and human cells. Mapping of the murine BMDC and human HEK293 cells dataset containing gene-specific RFP density values in biological duplicates to the stability profiling groups yielded totals of 2,321 and 2,736 genes, respectively. Finally, the intersection (*n* = 1,950 genes) between mouse BMDCs and human HEK-293 cells genes has been used for the comparative analysis (Supplementary Figures 2C,D), where the distribution of genes was as follows: rS-pS (*n* = 593), rS-pU (*n* = 682), rU-pS (149), and rU-pU (*n* = 526) (Supplementary Table 3). The comparison of average BMDC RFP density values in the four stability groups uncovered that the highest RFP density in the rS-pS group, while the lowest RFP density, was identified in the rU-pU group (Supplementary Figure 2C). Of note, the groups with stable mRNAs with or without stable protein have higher RFP density in comparison to the groups with unstable mRNAs, highlighting the correlation between mRNA stability and RFP density.

In human HEK293 cells, interestingly the difference among the group is quite striking and similar to that of murine BMDC cells (Supplementary Figure 2D). For example, the rS-pS group followed by the rS-pU group displayed a relatively higher average ribosome footprinting density, whereas the unstable mRNA and protein (rU-pU) group showed the lowest. The ribosome footprinting density of the genes with stable mRNA and protein (rS-pS group) was significantly higher compared to the rest of the groups highlighting how mRNA and protein stability contributes to the ribosome binding capacity and translational efficiency. This trend of higher-to-lower RFP density across the stability groups (rS-pS > rS-pU > rU-pS > rU-pU) was similar in both BMDC and HEK-293 cells.

### Conservation of Stability-Dependent mRNA-to-Protein Correlation Patterns Across Human Tissues

To explore the conservation of the impact of mRNA and protein stability on their correlation pattern in human tissues, we have obtained multiple transcriptome and proteome datasets across 36 human tissues. We constructed three independent datasets (Datasets 1, 2, and 3) by integrating genome-wide mRNA and protein abundances generated by RNA-seq and LC-MS/MS-based mass-spectrometry techniques, respectively. Dataset 1 (*n* = 14 tissues) comprised the intersect of the transcriptome and proteome datasets from GTEx (Consortium, 2013) and HPM (Kim et al., 2014), respectively (Supplementary Table 4). Dataset 2 (*n* = 12 tissues) and Dataset 3 (*n* = 29 tissues) incorporating transcriptome and proteome datasets were obtained from Wilhelm et al. (2014) and Wang et al. (2019), respectively (Supplementary Table 4). To explore the data variability originating from different experimental settings and/or technical issues that may potentially contribute to uncertainty in our multi-omics integration approach, we performed an extensive quality control analysis. To this end, we first compared the expression of mRNAs from three different datasets in all possible combinations (Dataset 1 vs. 2, Dataset 1 vs. 3, and Dataset 2 vs. 3) across seven overlapping tissues (adrenal gland, esophagus, kidney, ovary, pancreas, prostate, and testis) (Supplementary Figures 3A–C). These analyses showed that mRNA expression profiles were quite similar and showed a strong cross-correlation between datasets ranging from 0.95 to 0.81 with a mean correlation coefficient of 0.86. These results suggest that transcriptomes tend to correlate strongly independent of unmatched donor sampling. Next, we compared the protein expression from three different datasets in all possible combinations as described above across seven overlapping tissues. Similar to transcriptome analysis, we identified a strong correlation between the proteomes of different tissues ranging from 0.5 to 0.79 with a mean correlation coefficient of 0.66 (Supplementary Figures 3D–F). Overall these strong correlations of mRNAs and protein levels indicated that dataset-specific variability does not contribute significantly to the global variability of mRNA and protein correlation among three different datasets.

**Figure 2:**
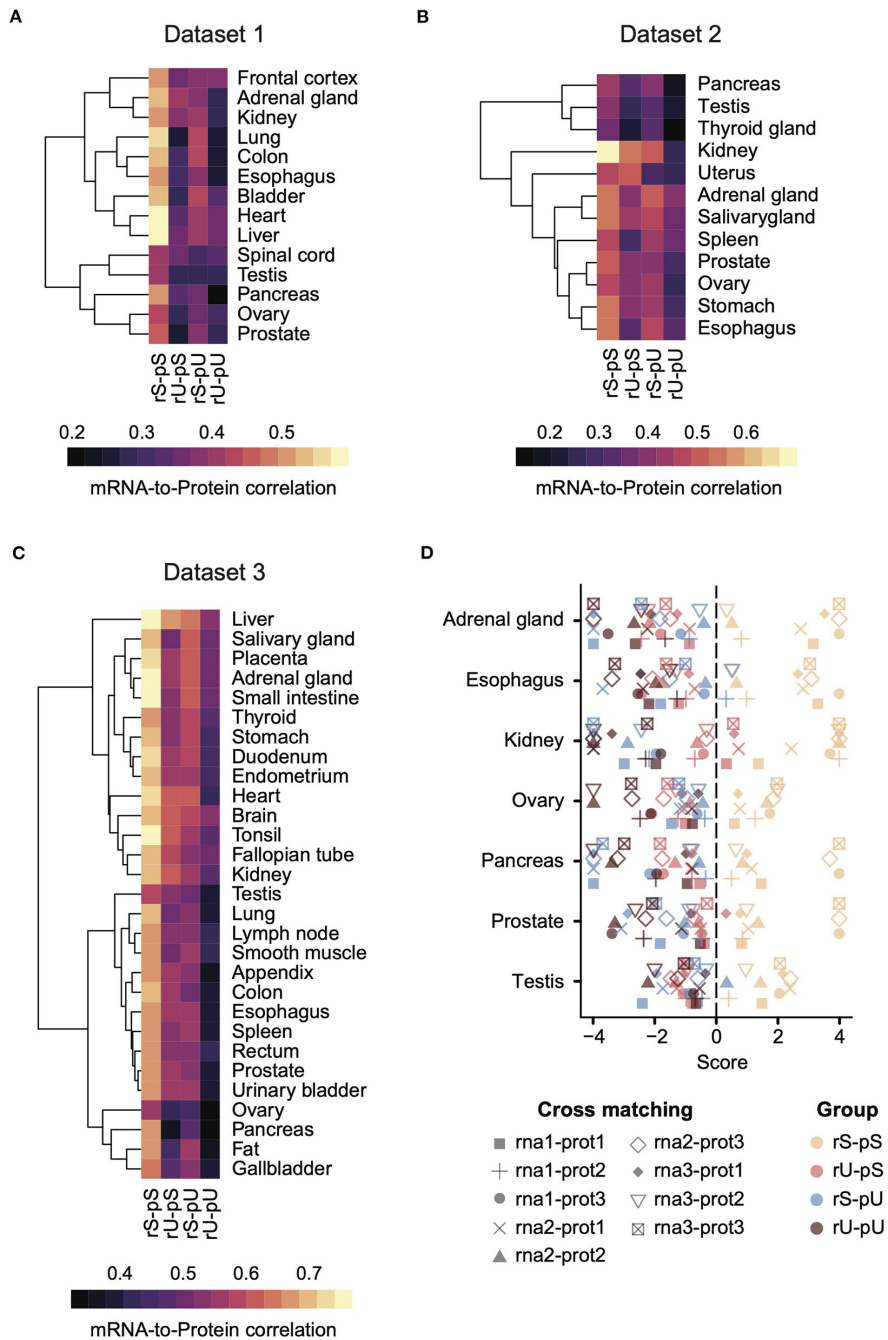
A conserved stability-dependent mRNA-to-protein correlation pattern in diverse human tissues. Heatmaps representing the mRNA-to-protein Spearman correlation coefficients in Dataset 1 **(A)**, 2 **(B)**, and 3 **(C)** across different tissues for each stability group. Each dataset comprised gene-matched mRNA and protein abundances obtained from independent sources. The color code was based on Spearman correlation coefficients. Rows indicating different tissues were clustered using hierarchical clustering based on Euclidean distance. **(D)** Cross-matching analysis between Datasets 1, 2, and 3 where the combinations of different mRNA and protein abundance are illustrated by different types of symbols. Color code represents the four stability groups. The score was defined as –log10 upper-tail *p*-value from empirical cumulative distribution when the upper-tail *p*-value was lower than the lower-tail *p*-value. Otherwise, the score was defined as +log10 lower-tail *p*-value.

Each dataset was then analyzed separately by estimating the mRNA-to-protein correlation per stability group in a tissue-specific manner (Figures 2A–C). An analysis of variance (ANOVA) test showed a significant variance across the stability groups (ANOVA *p* = 1.09 × 10^−14^, 5.23 × 10^−7^, <2 × 10^−16^ for Datasets 1, 2, and 3, respectively). A consistent pattern emerged across all the datasets, where the genes with stable (rS-pS) and unstable (rU-pU) mRNAs and proteins exhibited the highest and the lowest correlation, respectively, across all datasets irrespective of tissue types. The genes with mixed stability profiles (rU-pS and rS-pU) showed a heterogeneous correlation pattern across tissues and datasets. Nevertheless, the average correlation in rS-pU was always higher than in rU-pS, but this difference was significant only in Dataset 1 (two-sided *t*-test *p* = 0.003, 0.147, and 0.285 for Datasets 1, 2, and 3, respectively). We performed the bootstrap analysis (as described previously) to determine whether the stability-dependent correlation pattern in each stability group was significantly different (higher than random or lower than random) from what is expected by chance (Supplementary Table 9). This analysis demonstrated a significantly higher correlation in the rS-pS group in most of the tissues (liver, heart, colon, kidney, ovary, prostate, stomach, and testis). On the opposite, the rU-pU group had a significantly lower correlation than expected in all tissues except spleen tissue from Dataset 2.

To investigate whether the hierarchical pattern of stability-dependent mRNA-to-protein correlation is independent of the organization of paired transcriptome and proteome datasets, we performed a cross-matching analysis of these three datasets. Genes for which mRNA and protein abundances were not quantified in at least 1 dataset were excluded to make the datasets uniform. Next, we calculated the correlation between mRNAs and proteins across different datasets in all possible combinations (e.g., rna1-prot2 means mRNA and protein abundances from datasets 1 and 2, respectively) (Figure 2D). The mRNA-to-protein cross-matching analysis revealed a conserved hierarchical pattern of stability-dependent mRNA-to-protein correlation that we observed in individual datasets. Indeed, the stable group (rS-pS) showed the highest correlation score, where this score is equal to -log10 (upper-tail *p*-value) when upper-tail *p*-value < lower-tail *p*-value, or log10 otherwise. Altogether, these data suggest a conserved pattern across diverse human tissues (Figure 2D).

### Conservation of Stability-Dependent mRNA-to-Protein Correlation Patterns Across Cell Lines

**Figure 3:**
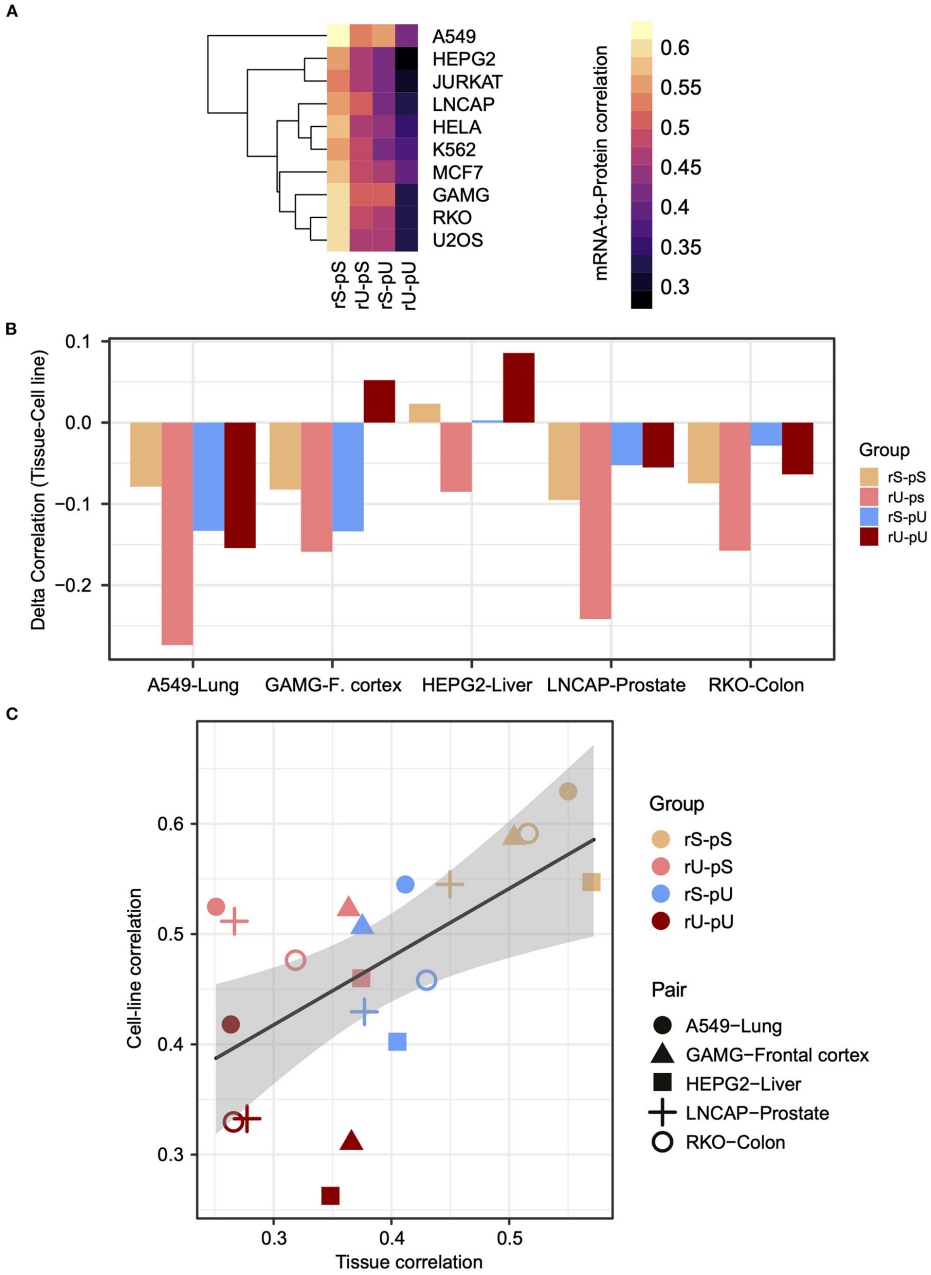
The stability-dependent mRNA-to-protein correlation pattern is consistent between tissues and matched cell lines. **(A)** Heatmap illustrating the mRNA-to-protein Spearman correlation across different cell lines. The color code indicates the range of the correlation coefficient values. Rows representing different cell lines were clustered using hierarchical clustering based on Euclidean distance. **(B)** Barplots showing the difference of mRNA-to-protein Spearman correlation (defined as delta correlation) between different cell lines and their matched tissues of origin. The color code indicates the stability groups. **(C)** Scatterplot demonstrating the relationship between mRNA-to-protein Spearman correlation coefficients in matched tissues (*X* axis) against cell lines (*Y* axis). The color codes represent different stability groups. The five matched tissue–cell line pairs are illustrated with a different type of symbols. R indicates Spearman correlation coefficient of the slope of the regression line. *p*-Value indicates the significance of correlation.

We hypothesized that the stability-dependent mRNA-to-protein correlation patterns should also be conserved in the human cell lines. We took advantage of the mRNA and protein datasets representing the transcriptome and proteome, of 11 most commonly used human cell lines from Geiger et al. (2012) to test the hypothesis. To make the cell line dataset consistent with the tissue, the genes from the cell line dataset that were also quantified in the tissues were selected (Supplementary Table 5). The results demonstrated that the dependency of the mRNA-to-protein correlation on their respective stability was conserved in cell lines as the highest and lowest mRNA-to- protein correlations were again observed for the stable (rS-pS) and unstable (rU-pU) groups, respectively (Figure 3A). In case of the mixed stability groups, rU-pS showed a relatively high correlation compared rS-pU group (Figure 3A) (two-sided *t*-test *p*-value = 0.056). To directly compare tissues with cell-line correlations, five cell lines (A549, GAMG, HEPG2, LNCAP, and RKO) were matched with the tissues based on their cellular origin to construct five tissue–cell line pairs (A549–lung, GAMG–frontal cortex, HEPG2–liver, LNCAP–prostate, and RKO–colon) followed by the comparison of mRNA-to-protein correlation. A significantly higher correlation in cell lines was identified compared to tissues (two-sided *t*-test *p* = 0.014), which was reflected by the negative correlation difference between a tissue–cell line pair (Figure 3B and Supplementary Table 10). Interestingly, the highest correlation difference was observed for the rU-pS group. There were only two instances where the higher correlations in tissues were observed for the liver and frontal cortex (Figure 3B and Supplementary Table 10). However, the stability group–specific correlation-differences were not significant in any tissue–cell line pair as revealed by bootstrap analysis. Next, we asked whether the mRNA-to-protein correlation values are influenced by the tissue/cell line origin or depend on stability profiling. The results indeed showed that the tissue origin does not have a significant impact; rather, the correlation values between paired tissues and cell lines were distributed according to their stability groups (Figure 3C). For instance, the stable (rS-pS) group showed the highest correlation values irrespective of their tissue origin. Similarly, the lowest correlation values were observed for the unstable (rU-pU) group. Overall, the stability group–dependent correlation values were found to be positively correlated (*r* = 0.57, *p* = 0.003) across tissue–cell line pairs (Figure 3C). In summary, these results emphasize a conserved stability group–specific mRNA-to-protein correlation in tissues and derived cell lines.

### RNA-to-Protein Correlation Alteration in Tumor Tissue

Having established the conservation of stability-dependent correlation patterns across human tissues and cell lines, we asked whether any deviation in the mRNA-to-protein correlation occurs in a cancer state. To this aim, we retrieved the transcriptome and proteome data of COAD samples from TCGA (Zhang et al., 2014) and CPTAC, respectively (Edwards et al., 2015) (Supplementary Table 6). We have chosen this particular cancer type because of its high sample rate and availability of proteogenomics data facilitating the statistical analysis. The same stability-dependent correlation patterns were emerged, thus further extending the hypothesis that the impact of stability is conserved in the altered cellular state, such as cancer (ANOVA *p* < 2 × 10^−16^) (Supplementary Figure 4A). The patient-wise analysis also corroborated this conserved pattern of stability-dependent correlation patterns (Supplementary Figure 4B) showing the highest correlation in the rS-pS group followed by rU-pS and rS-pU, whereas the lowest correlation was observed for rU-pU across all the patients. This remarkable conservation of stability-dependent correlation patterns in a patient-wise manner greatly strengthens our hypothesis that the pattern is conserved irrespective of individual transcriptome and proteome variability.

**Figure 4:**
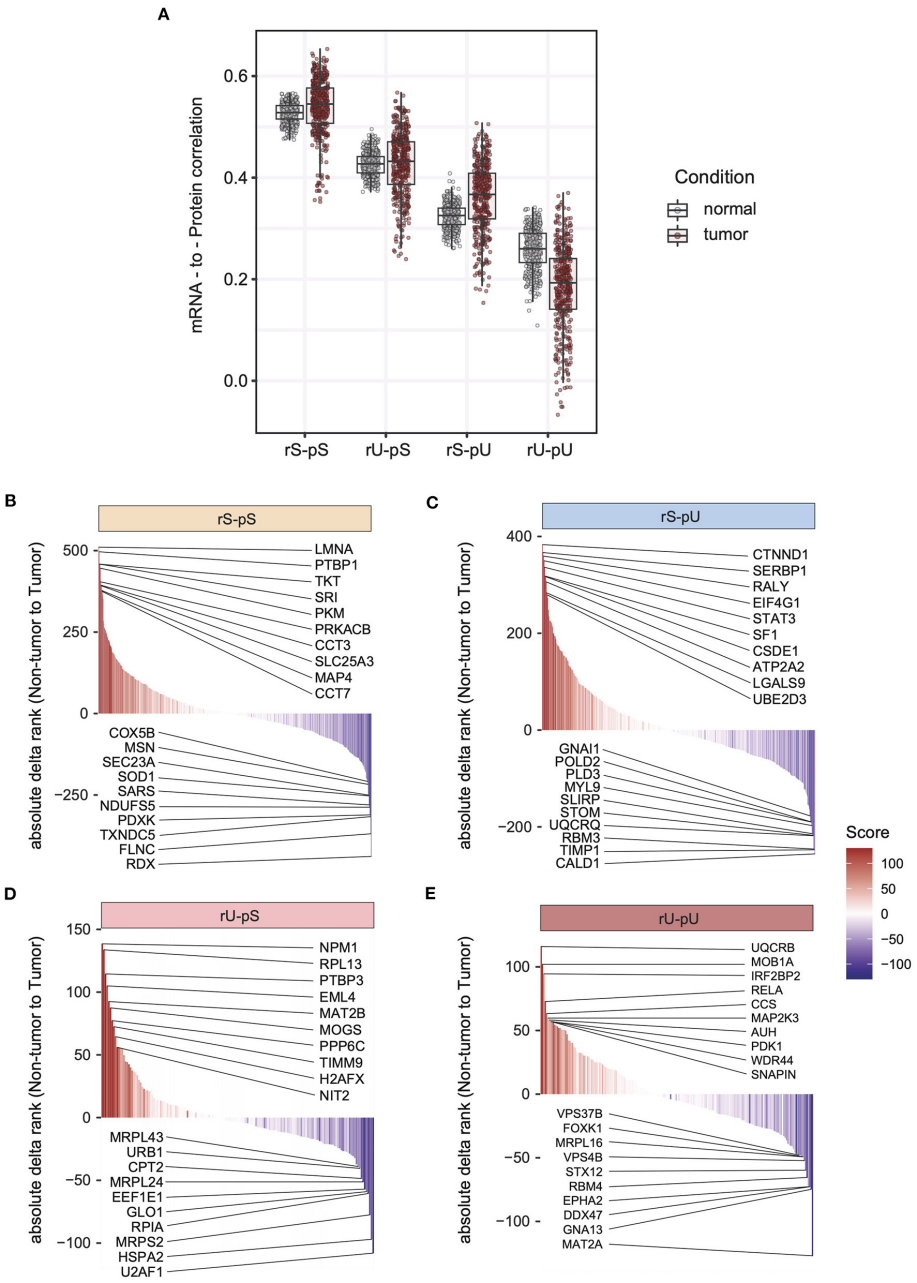
Altered stability-dependent mRNA-to-protein correlation pattern in tumors compared to non-tumor tissues. **(A)** Distribution of the mRNA-to-protein correlation obtained in an unpaired analysis visualized as a boxplot showing the median and interquartile range of mRNA-to-protein correlation (Spearman correlation) in each stability group. Each dot represents an mRNA-to-protein correlation obtained from a single mRNA/protein combination of tumor or non-tumor samples among the 400 combinations that were tested. Color code indicates the tumor or non-tumor tissues. The cumulative rank plots highlighting the difference of absolute delta rank (mRNA—protein) in non-tumor vs. tumor samples are for rS-pS **(B)**, rS-pU **(C)**, rU-pS **(D)**, and rU-pU **(E)** groups. The color code represents the rank difference between non-tumor and tumor. Red color indicates a higher rank in tumor, and blue colon indicates a lower rank in the tumor (higher rank in non-tumor). The top 10 correlation-enhancing (red) and -repressing (blue) genes were labeled for each stability group.

**Figure 5:**
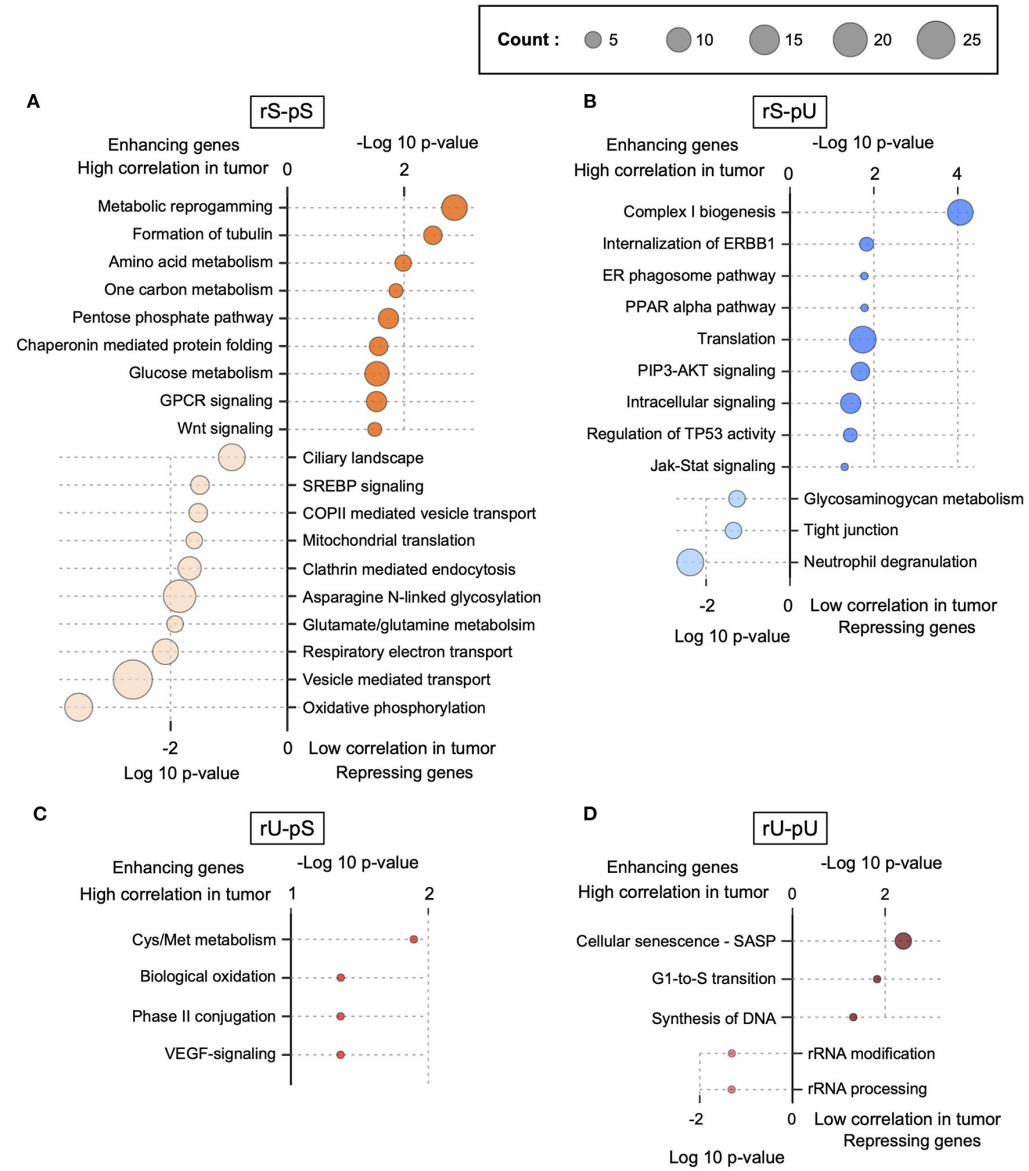
Functional characterization of correlation-enhancing and -repressing genes. Enriched terms of ConsensusPathDB, obtained by enrichment analysis of correlation-enhancing and -repressing genes in rS-pS **(A)**, rS-pU **(B)**, rU-pS **(C)**, and rU-pU **(D)** are shown. *p*-Values implying the significance from Fisher exact test are shown either as –log10 *p*-value for pathways associated with correlation-enhancing genes or +log10 *p*-value for pathways associated with correlation-repressing genes. The size of the bubble is proportional to the number of annotated genes identified for a particular pathway within the enhancing or repressing gene lists.

The lack of patient-matched proteome data in non-tumor adjacent colon tissues was compensated by obtaining proteome data for non-tumor healthy colon tissues (*n* = 30) from CPTAC. The lack of patient-matched datasets compelled us to adopt an unpaired correlation analysis. To this end, we randomly selected 20 samples from each of the non-tumor RNA, non-tumor protein, colon tumor RNA, and colon tumor protein datasets. The mRNA-to-protein correlation was calculated for all the possible combinations between RNA and protein in tumor and non-tumor tissues. In total, 800 (400 each) correlation values were obtained in tumor and non-tumor colon tissues. Interestingly, the overall correlation is higher in tumors compared to non-tumor tissues (two-sided Wilcoxon test *p* = 4.74 × 10^8^) (Supplementary Figure 4C). The mRNA-to-protein correlations in three stability groups (rS-pS, rU-pS, and rS-pU) were increased, whereas surprisingly, the correlation in rU-pU was decreased in tumor compared to non-tumor tissues (Figure 4A). Next, we performed the bootstrap analysis to assess the stability group–specific variation of correlation between tumor and non-tumor tissues. Interestingly, the Wilcoxon rank test revealed that the correlation in the non-tumor samples was significantly higher compared to tumor samples in the rU-pU group (two-sided Wilcoxon rank test *p* = 0.038) (Figure 4A and Supplementary Figure 4D). A lower correlation may emerge from different origins including the rapid turnover of the mRNAs and/or proteins for these genes or simply from the increased noise in their abundances in cancer tissue. Next, we assumed that we may learn more by analyzing the difference of correlation between stability groups. Indeed, this analysis revealed that the correlation difference between rS-pU and rU-pU in tumors was significantly larger than that of non-tumor tissues (*p* = 0.036) (Supplementary Figure 4E). Following a similar trend, the correlation difference between rS-pS and rU-pU groups was also found to be marginally significant (*p* = 0.057) in tumors compared to non-tumor tissues (Supplementary Figure 4E). To further shed light on this interesting pattern, it is of high interest to analyze the correlation in a gene-centric manner to identify the genes that are driving the correlation deviation in tumors. For this purpose, we investigated the difference of correlation rank between mRNAs and proteins in tumor and non-tumor tissues. As our correlation analysis is based on rank, a similar rank in mRNA and protein tends to increase the absolute correlation. On the opposite, the big difference between ranks tends to decrease the absolute correlation. By comparing the absolute difference of mRNA and protein ranks in tumor and non-tumor tissues, genes were classified as tumor correlation-enhancing or -repressing genes. Tumor correlation-enhancing genes represent all the genes that contribute to increase the absolute correlation, whereas correlation-repressing genes contribute to decrease the absolute correlation in tumor compared to non-tumor tissue. Tumor correlation-enhancing and -repressing genes are shown in red and blue, respectively, for rS-pS (4B), rS-pU (4C), rU-pS (4D), and rU-pU (4E) in Figures 4B–E. Some interesting genes were identified as correlation-enhancing genes—*PKM*: pyruvate kinase and repressing genes; *COX5B*: cytochrome c oxidase subunit 5B in rS-pS (Figure 4B)—indicating a possibility of alterations of mRNA-to-protein correlation of the energy metabolism–associated genes in the tumor. Moreover, TFs, such as STAT3 in rS-pU and RELA in rU-pU were identified as correlation-enhancing genes (Figures 4C,E). The next question was whether these tumor correlation-enhancing and -repressing genes represent specific biological pathways that are associated with tumor progression. We investigated these genes by Fisher exact test to identify the enriched biological pathways. Indeed, certain metabolic and cancer-associated signaling pathways were enriched for different stability groups (Figure 5). For instance, metabolic reprogramming, pentose–phosphate pathway (PPP), and WNT-signaling pathway were enriched (as indicated by –log10 *p*-values) in the correlation-enhancing gene sets, while oxidative phosphorylation and respiratory electron transport chain were enriched (indicated by log10 *p*-values) in the correlation-repressing gene set of the rS-pS group (Figure 5A). For the rS-pU group, the enriched pathways in correlation-enhancing gene sets include complex I biogenesis, Endoplasmic Reticulum (ER)–phagosome pathway, translation, PIP3-AKT, and JAK-STAT signaling while neutrophil degranulation and tight junction were associated with correlation-repressing gene set (Figure 5B). In the rU-pS group, enriched pathways—biological oxidation and VEGF-signaling were only identified to be associated with correlation-enhancing genes while no enriched pathways were identified for correlation- repressing genes (Figure 5C). Lastly, in the rU-pU group, cellular senescence-associated secretory phenotype (SASP), G1-S transition, and synthesis of DNA were associated with correlation-enhancing genes while the rRNA associated pathways were identified for correlation-repressing genes (Figure 5D).

**Figure 6:**
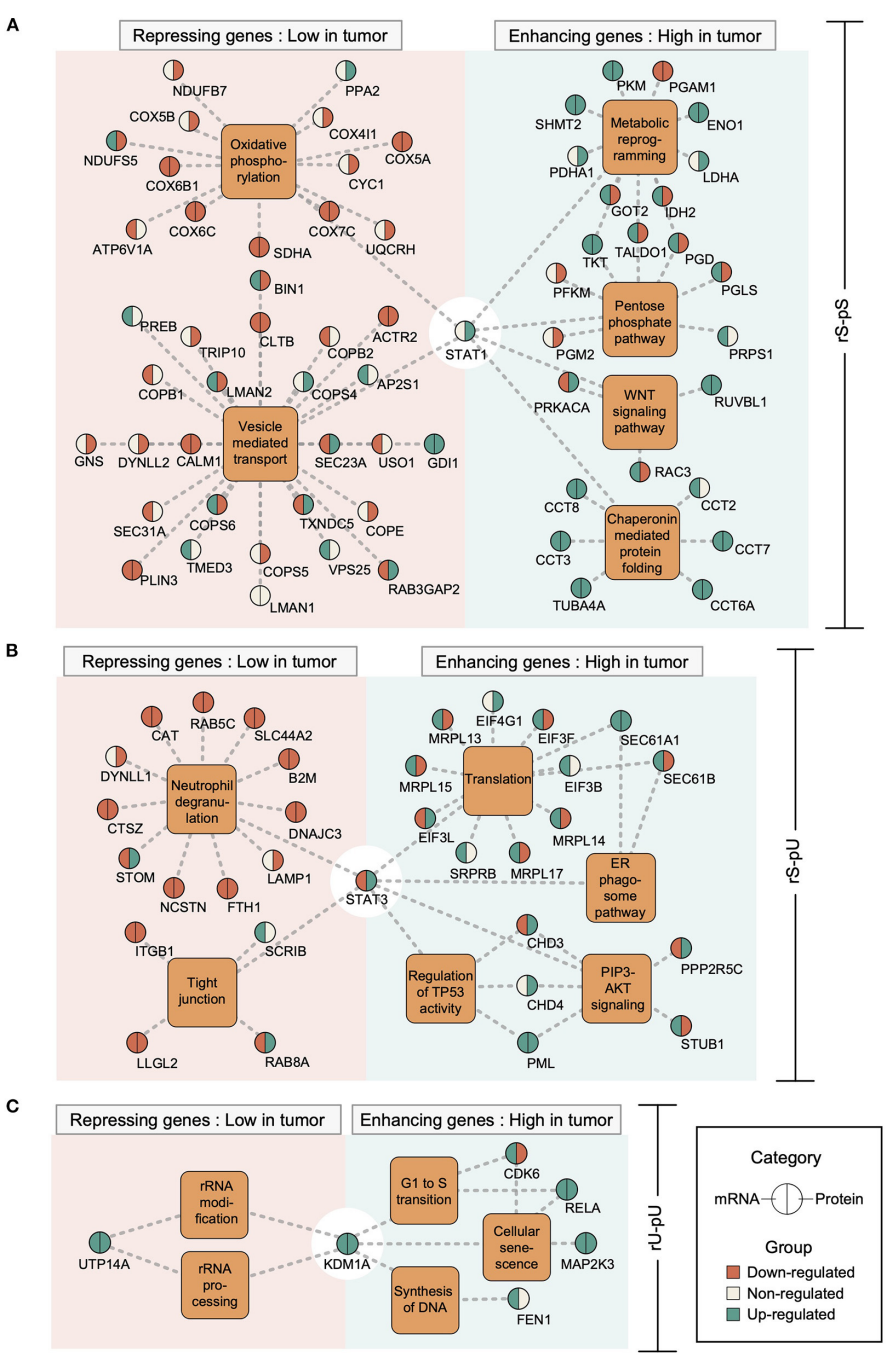
Transcriptional regulatory networks controlling the correlation-enhancing and -repressing genes. Transcription factor (TF)–target gene network was constructed based on Chip-seq data from ENCODE representing the TF–target gene relationship. The different TF–target gene networks: STAT1 network for rS-pS **(A)**, STAT3 network for rS-pU **(B)**, and KDM1A network for rU-pU **(C)** are shown. TFs are connected to their known targets correlation-enhancing and -repressing genes through the enriched pathways from Figure 5. Color code indicates the regulatory pattern of mRNAs/proteins in tumor tissues (green indicates upregulation in tumor, whereas red indicates downregulation in the tumor) as defined by the *p*-value (*p* < 0.05 was set as significant and considered as regulated). The left and right parts of the nodes delineate mRNA and protein regulations.

Previously it has been shown that the mRNA regulation through transcriptional regulatory circuits is the most dominant factor, to control the corresponding protein levels to carry out a specific function in an altered cellular state (Jovanovic et al., 2015). In light of this assumption, we hypothesized that in a cancer state some transcriptional regulatory network may contribute to the altered mRNA-to-protein correlation by regulating the mRNA levels which in turn may determine the corresponding protein levels. To test our hypothesis, we retrieved the association between TFs and their target genes from ENCODE (Consortium, 2011) followed by the visualization of the TF–target gene network by using Cytoscape (Shannon et al., 2003). Among the six identified TFs, those with significantly high or low mRNA-to-protein correlation and with differential mRNA and/or protein levels between tumor and non-tumor tissues were considered (Supplementary Figures 5A–F). In total, five TFs have fulfilled the criteria: STAT1 (rS-pS), STAT3 and PML (rS-pU), KMD1A, and RELA (rU-pU) (Supplementary Figures 5A–E). Another TF—TBL1XR1 targeting rU-pS genes was not considered for network analysis since it's mRNA and corresponding protein levels were not significantly regulated between tumor and non-tumor tissues (Supplementary Figure 5F). The TF–target gene network analysis uncovered an unexpected scenario where both correlation-enhancing genes (with higher correlation in the tumor) and -repressing genes (with lower correlation in the tumor) were targeted by STAT1 (6A), STAT3 (6B), KDM1A (6C), PML (Supplementary Figure 6A) and RELA (Supplementary Figure 6B). STAT1 appears to positively control the correlation-enhancing genes associated with metabolic reprogramming, PPP, WNT-signaling, and chaperon mediated protein folding (Figure 6A). The mRNA levels of most of the correlation-enhancing genes under STAT1 control were upregulated in tumors compared to non-tumor tissues. In contrast, STAT1 seems to exert negative control over correlation-repressing genes belonging to oxidative phosphorylation and vesicle-mediated transport by downregulating most of their mRNA and protein levels (Figure 6A). STAT3 and PML were found to exert positive control over the upregulation of most of the mRNA levels of correlation-enhancing genes comprising translation, ER–phagosome, TP53 regulation, and PIP3-AKT signaling. In contrast, STAT3 appears to repress the mRNA levels of most of the correlation-repressing genes of neutrophil degranulation and tight junction pathways (Figure 6B and Supplementary Figure 6A). Similarly, two TFs, KDM1A, and RELA, exhibited a positive effect on the mRNA levels of genes associated with G1-S transition, SASP, and DNA synthesis while showing a negative effect on rRNA modification and processing (Figure 6C and Supplementary Figure 6B). In summary, we functionally characterized the correlation-enhancing and -repressing genes and highlighted the transcriptional regulatory circuits that may be involved in simultaneous positive and negative regulation of correlation-enhancing and -repressing genes, respectively, belonging to diverse metabolic and cancer-associated pathways.

We also investigated the influence of age, gender, histologic subtype, and tumor location on the mRNA-to-protein correlation. Previously, it has been assumed that the molecular profiles, such as higher frequencies of microsatellite instability–high, PTEN, and HRAS mutations, may occur in colorectal cancer patients with age <50 years than those of higher age groups (Chang et al., 2017). In light of this finding, we aim to explore the impact of age on the mRNA-to-protein correlation in colon cancer patients by classifying the patients older or younger than 50 years. The analysis revealed that patients <50 and >50 years old showed no significant difference in the mRNA-to-protein correlation (Supplementary Figure 7A). Similarly, gender appeared to have no influence on the mRNA-to-protein correlation in COAD tissues (Supplementary Figure 7B). Next, we investigated the impact of tumor locations (ascending colon, cecum, colon-Not Otherwise Specified (NOS), descending colon, rectosigmoid junction, sigmoid colon, transverse colon) on the mRNA-to-protein correlation. The results showed that high variability among the different tumor-location groups and no-significant deviations was observed in the mRNA-to-protein correlation values (Supplementary Figure 7C). Additionally, we tested the impact of the histologic subtypes (adenocarcinoma-NOS and mucinous adenocarcinoma) on mRNA-to-protein correlation, but no significant effect was observed (Supplementary Figure 7D). Another important clinical feature that may directly have an impact on mRNA-to-protein correlation is tumor purity. It has been previously shown that tumor purity can vary significantly among the TCGA tumor samples (Aran et al., 2015). Tumor purity of the 58 COAD samples was assessed by analyzing IHC result (estimated by image analysis of hematoxylin and eosin–stained slides) and CPE (Aran et al., 2015). COAD patients were subsequently divided into three groups (high, intermediate, and low) based on the tumor purity scores (ICH and CPE) using three-quantiles. The mRNA-to-protein correlations were then compared among the different purity groups. Interestingly, no significant deviation was observed among the different purity groups (Supplementary Figures 8A,B).

In addition to clinicopathological features, it is important to explore the possible impact of the tumor molecular features (e.g., proliferation, hypoxia, and EMT) on mRNA-to-protein correlation. To this end, we first identify the molecular features specific to COAD by doing a single-sample hallmark gene-set enrichment analysis on COAD transcriptome data. The patients were subsequently classified into three different groups (high, intermediate, and low) based on the NES. Interestingly, mRNA-to-protein correlation values exhibited a significant positive correlation for one particular feature—mitotic spindle, in which patients with higher mitotic spindle assembly showed a higher mRNA-to-protein correlation compared to patients with lower ones (two-sided Wilcoxon test *p* = 0) (Supplementary Figure 8C). Other molecular features, such as EMT and hypoxia did not significantly correlate with mRNA-to-protein correlation among the COAD patients (Supplementary Figures 8D,E).

### Differential mRNA-to-Protein Correlation Pattern Between Early and Late Stage of Tumor

**Figure 7:**
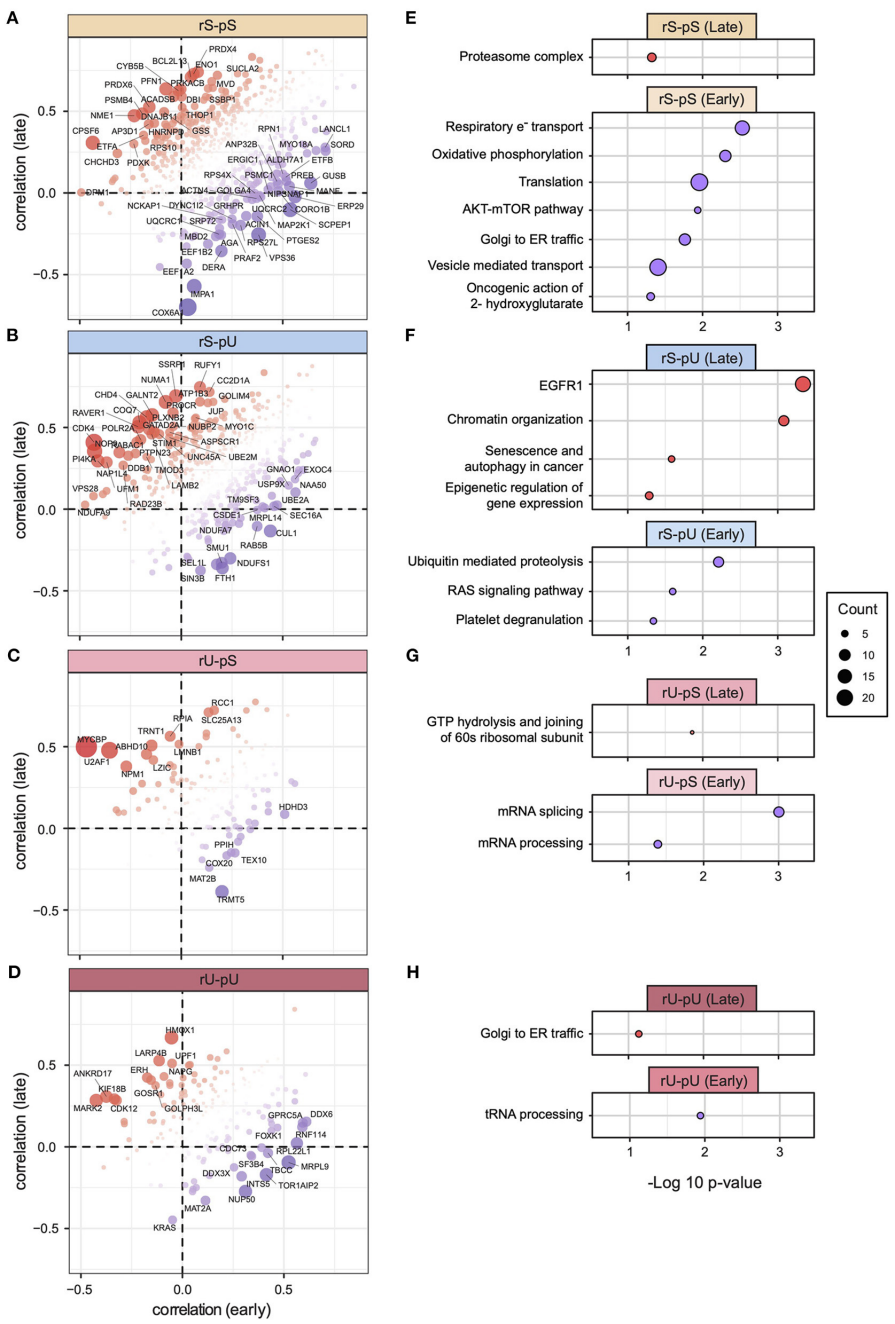
Gene-centric mRNA-to-protein correlation in early vs. late phases of COAD. Scatterplots representing the gene with most deviating mRNA-to-protein correlation between early and late phases (with a cutoff *p* < 0.05) for each stability group are shown: **(A)** rS-pS (*n* = 63), **(B)** rS-pU (*n* = 41), **(C)** rU-pS (*n* = 16), **(D)** rU-pU (*n* = 26). Color code and bubble size represent the difference in correlation and *t*-test *p*-values, respectively. Enriched terms of ConsensusPathDB with *p* < 0.05 that are associated with the most deviating genes between early and late phases are illustrated for each stability group: **(E)** rS-pS, **(F)** rS-pU, **(G)** rU-pS, **(H)** rU-pU. The total number of genes that were used for enrichment analysis in each stability group is as follows: rS-pS (*n* = 63), rS-pU (*n* = 41), rU-pS (*n* = 16), and rU-pU (*n* = 26). Significance from Fisher exact test is shown as –log10 *p*-value. The size of the bubbles is proportional to the number of annotated genes associated with a particular consensus term within the gene list with significant deviating mRNA-to-protein correlation.

The 58 tumor samples from patients different with clinical stages (I, II, III, and IV) were divided into early (combined stage I and II, *n* = 32) and late (combined stage III and IV, *n* = 26) stages to make the stage-specific dataset comparable in terms of sample size (Supplementary Table 7). As both mRNA and protein datasets were available for the early- and late-stage tumor samples, we conducted a patient-matched paired analysis resulting in mRNA-to-protein correlation values from early- and late-stage patients. We calculated the correlation per stability group and compared the early- and late-stage correlations. A two-tailed *t*-test did not show any significant differences between the tumor stages (Supplementary Figure 9A). Although the stability group-wise correlations did not deviate significantly, a gene-centric correlation analysis identified the genes with deviating mRNA-to-protein correlations between early and late stages in rS-pS (*n* = 63) (Figure 7A), rS-pU (*n* = 41) (Figure 7B), rU-pS (*n* = 16) (Figure 7C), and rU-pU (*n* = 26) (Figure 7D) groups. In the rS-pS group, some interesting genes with deviating correlation between early and late stages including *PSMB4* (proteasome subunit beta type-4) with high correlation in the late stage and *EEF1B2* (elongation factor 1-beta) with high correlation in the early stage of tumor tissue were identified. Another interesting gene in the rS-pS group *COX6A* (cytochrome c oxidase subunit 6A1) (mitochondrial), the last enzyme in the mitochondrial electron transport chain, exhibited a very low correlation in the late tumor stage compared to the early stage. For the rS-pU group, *CHD4* (chromodomain-helicase-DNA–binding protein 4) and *GATAD2A* (transcriptional repressor p66-alpha) were found to be highly correlated in the late stage, whereas *UBE2A* (ubiquitin-conjugating enzyme E2A) that plays a role in the regulation of histone proteins by ubiquitin-mediated proteolysis was highly correlated in the early stage. rU-pS gene *PPIH* (peptidyl-prolyl *cis*-*trans* isomerase H) that participates in pre-mRNA splicing and rU-pU gene *NUP50* (nuclear pore complex protein Nup50) involved in nuclear protein import were highly correlated in the early stages. The previously identified TFs—STAT1, STAT3, KDM1A, PML RELA, and TBL1XR1—did not exhibit a deviating correlation between the early and late tumor stages (Supplementary Figures 9B–G), indicating that their correlation remains constant throughout the different stages of the tumor compared to non-tumor tissues. To functionally characterize the genes with deviating mRNA-to-protein correlations, a Fisher exact test revealed the pathways encompassing the genes that showed a significantly higher correlation in the early or late tumor stages across the stability groups. In the rS-pS group, genes associated with oxidative phosphorylation and electron transport chain showed a higher correlation in the early stages (lower correlation in late stages), and proteasome complex exhibited a higher correlation in the late stage (Figure 7E). These results indicate that the metabolic reprogramming regulatory circuit may not be fully established at the early stages but rather emerged through the transition from early to late stages. In the rS-pU group, EGFR signaling and chromatin organization–associated genes showed a higher correlation in the late stage, whereas ubiquitin-mediated proteolysis genes showed a higher correlation in the early stage (Figure 7F). Genes comprising mRNA and tRNA processing pathways exhibited a higher correlation in the early stages of COAD (Figures 7G,H).

## Discussion

The current article attempted to show that the mRNA-to-protein correlation analysis can serve as a tool to identify cancer-specific transcriptional pathways that are necessary for cancer progression by using COAD as a model dataset. Through the current study, we gained important insight into a fundamental aspect of mammalian proteotranscriptomic landscape by providing compelling evidence that genome-wide mRNA-to-protein correlation depends on their stability. Results suggest that a hierarchical correlation pattern, constrained by the stability of mRNA and protein molecules, is conserved across mammalian species irrespective of the tissue types. We harnessed the stability-dependent correlation patterns to further shed light on the alteration of gene-centric mRNA-to-protein correlation and identified correlation-enhancing and -repressing genes in the tumor tissues. Furthermore, we uncovered the transcriptional regulatory circuits controlling the correlation-enhancing and -repressing genes that are associated with metabolic reprogramming and cancer-associated pathways in tumor tissues. We uncovered the gene-specific differential mRNA-to-protein correlation pattern in the early and late stages of tumor tissues. The results showed a gene-specific differential mRNA-to-protein correlation between the early and late stages of tumor highlighting the possibility that the tumor cells control different pathways in different clinical stages by tightly regulating the corresponding mRNA and protein levels of different genes. By tightly controlling the mRNA and corresponding protein levels of the target genes, hence their correlation, the transcriptional regulatory circuits may enable the cancer cells to mitigate the challenges posed by the tumor microenvironment. Finally using the mRNA-to-protein correlation profiling, we identified different pathways that are differentially controlled by the cancer cells, in the early and late stages of the tumor. Furthermore, investigation of the impact of age, gender, histologic subtype, and tumor location revealed that mRNA-to-protein correlations alterations do not depend on the clinicopathological and demographic parameters but rather emerge from the different molecular processes including mRNA and protein stability and production rates. Previously, it has been proposed that dysregulation of mitotic spindle formation may contribute to chromosomal instability in colon cancer (Dalton and Yang, 2007). Interestingly, a positive correlation was observed between the mitotic spindle formation and the mRNA-to-protein correlation among the COAD patients implying that the mRNA-to-protein correlation maybe influenced by the proliferative state where a higher proliferative state may increase the global mRNA-to-protein correlation (Supplementary Figure 8).

In this study, we not only revealed the quantitative relationship between mRNA-to-protein correlation and their stability profiles but also interestingly uncovered the conserved pattern of stability-dependent correlation across mammalian species including a diverse range of human tissues and cell lines. As RFP densities can be interpreted as translational efficiency (Jovanovic et al., 2015), the similar distribution of RPF density values across stability groups in both mouse and human cells underscores that the translational efficiency is most likely to be conserved across mammalian species. In light of these results, it is reasonable to assume that stability-dependent translational efficiency is likely to be an inherent property of the mammalian proteotranscriptomic landscape. This conserved nature of the interconnected stability and correlation profiles of mRNAs and proteins highlights the universal rates of mRNA–protein synthesis and degradation in different tissues and mammalian species. Apart from the conserved sequence features of mRNA and proteins controlling the key metabolic and cellular functions, the highly conserved nature of mRNA-degradation (Wilusz et al., 2001) and ubiquitin–proteasome system (UPS)–mediated protein degradation pathways (Zuin et al., 2014) may shape the evolutionarily conserved nature of mRNA and protein stability, which in turn manifest as the conserved stability-dependent mRNA-to-protein correlation pattern in mammalian systems.

The modest correlation observed between mRNAs and proteins in a global steady state may stem from the intrinsic (stochastic nature of the biochemical processes) and extrinsic noise (fluctuations of external conditions) in gene expression (Lin and Amir, 2018). In contrast to the steady state, the cellular response to an environmental challenge involves proteome adaptation guided by the transcription and translation of mRNAs and thereby reducing the noise in gene expression. Indeed Jovanovic et al. (2015) showed that, in a steady-state condition, mRNA levels of unstimulated mouse DCs contributed up to 68% to overall protein expression, whereas upon stimulation, changes in mRNA abundance of the genes associated with immune response pathways played an even more dominant role (up to 92%) and thus reducing the noise of gene expression. Interestingly, pathways other than the immune response relying on the preexisting proteins to perform the basic cellular functions were predominantly regulated at the level of protein translation or degradation (Jovanovic et al., 2015). Following the same line of argument, Koussounadis et al. (2015) provided a crucial piece of evidence stating that differentially expressed mRNAs tend to have a higher correlation with their corresponding protein product in comparison to the non-differentially expressed mRNAs in an ovarian cancer xenograft model. All this evidence leads to the hypothesis that adaptation of mammalian cells in response to the altered environment may require reducing the noise of gene expression that is reflected by the higher mRNA-to-protein correlation for the genes essential for the adaptation. In light of this hypothesis, we utilized the stability-dependent correlation pattern to quantitatively probe into the correlation-altering genes and associated biological pathways that are required for tumor cell development and transition from early to late stage of cancer. Indeed, we identified correlation-enhancing and -repressing genes and their associated pathways in tumor cells. The observed global increase in the mRNA-to-protein correlation in tumor tissues compared to non-tumor counterparts may reflect the global noise reduction in gene expression in cell lines and tumor tissues. However, the tumor tissues exhibited more variability in their mRNA-to-protein correlation compared to non-tumor tissues (Supplementary Figure 4C). Previously, Aran et al. (2017) showed that tumor tissue exhibits more variability in their mRNA expression compared to normal and adjacent non-tumor tissue. It has been proposed that intratumor heterogeneity is the underlying cause of the variability in mRNA expression in tumor tissues (Sun and Yu, 2015).

Previously, it has been proposed that noise in gene expression originating from the stochasticity of cellular processes gives an advantage to mammalian cells in terms of plasticity and flexibility by which cells can adapt to a new environment when it is required (Holmes et al., 2017). However, when cells respond to external stimulation or internal genomic alterations, the noise reduction for the genes that are required to cope with the altered environment comes at an energetic cost (Raser and O'Shea, 2005; Yan et al., 2016; Urban and Johnston, 2018). Following the same argument, in our study, correlation-enhancing genes exhibited a reduced noise in their gene expression as reflected in their higher mRNA-to-protein correlation, whereas correlation-repressing genes were noisy in their gene expression as evident by their lower mRNA-to-protein correlation.

To contextualize the biological interpretation, we identified the pathways associated with correlation-enhancing and -repressing genes. Energy metabolism pathways including glucose metabolism and metabolic reprogramming were associated with correlation-enhancing genes, whereas respiratory electron transport chain and oxidative phosphorylation were linked to the correlation-repressing genes in the tumor tissues compared to non-tumor tissues in the rS-pS group (Figures 5A, 6A). The reprogramming of energy metabolism pathways in colon cancer has been previously reported by several studies (Satoh et al., 2017; Brown et al., 2018; Zhang et al., 2019). When we investigated the gene-centric correlation alterations between early and late stages of tumor tissues, surprisingly the genes associated with oxidative phosphorylation and electron transport chain were highly correlated in early stages, while their correlation decreased dramatically in late stages (Figure 7E), thereby underscoring a stage-dependent regulation of these pathways. Moreover, the genes linked with glucose metabolism shared a similar correlation pattern in both stages. Earlier, it has been proposed that colon cancer may not be under the purely hypoxic condition as characterized by the “Warburg” phenotype; rather, the oxidative phosphorylation in colon cancer may serve as the main source of energy and even maybe upregulated in the early stage (Kaldma et al., 2014). Corroborating this, we extended the observation by providing evidence that oxidative phosphorylation and electron transport chain are most likely to be regulated in the early stage by transcriptional control, whereas in the late stage, these pathways may be under negative or no transcriptional control. A disrupted regulatory network of vesicle-mediated transport pathway associated with the correlation-repressing genes in the rS-pS group (Figures 5A, 6A) has been implicated in tumorigenesis (Tzeng and Wang, 2016). Interestingly, although the electron transport chain–associated genes were identified as correlation-repressing in the rS-pS group, Complex I (the largest complex of the mitochondrial electron transport chain) biogenesis was found to be enriched with correlation-enhancing genes (rS-pU group) in the tumor tissues (Figure 5B). Although this result may appear to be contradictory, recent evidence suggests the non-energetic roles of Complex I including cancer proliferation and metastasis (Urra et al., 2017), implying that Complex I biogenesis may provide an advantage to cancer cells in a non-energetic way. The rS-pU genes linked with the translational process exhibited correlation-enhancing property in the tumor compared to non-tumor tissues (Figures 5B, 6B). Furthermore, the mRNA-to-protein correlation pattern of the translational pathway–associated genes was not constant across different stages of cancer and appeared to be highly correlated in the early phase but not in the late stage of the tumor (Figure 7E). In contrast, proteasome complex genes showed a higher correlation in the late phase, signifying the role of the UPS in the advanced stages of the tumor (Figure 7E). Considering the importance of UPS in cancer progression and prognosis, different components of the UPS are now being considered as promising candidates for the treatment of cancer (Zhang et al., 2020). Regulation of TP53 activity associated with correlation-enhancing genes in tumor that comprised *CHD3* and *CHD4*–chromodomain-helicase-DNA–binding proteins (Figure 6B) was previously shown to downregulate TP53 levels by suppressing TP53 acetylation (Hirota et al., 2019). Higher mRNA-to-protein correlation driven by the transcriptional control of the genes downregulating TP53 activity may allow the tumor cells to evade the tumor-suppressor activity. Neutrophil degranulation linked to correlation-repressing genes in the rS-pU group (Figures 5B, 6B) represents another important pathway for cancer progression. Tumor-associated neutrophils were shown to have both protumorigenic and antitumorigenic activities, based on tumor diversity, tumor microenvironment, and the presence of an array of immune-modulating factors (Oberg et al., 2019). Similarly, correlation-repressing genes RAB8A and LLGL2 associated with tight junction (Figure 6B) have previously been shown to be downregulated in colorectal cancer (Spaderna et al., 2008; Letellier et al., 2017). In the rU-pU group, correlation-enhancing genes were found to be linked with G1 to S transition, DNA synthesis, and SASP (Figures 5D, 6C). SASP is characterized by a distinct secretory phenotype of senescent cells that may promote local inflammation and tumor metastasis (Sun et al., 2012). These early- and late-stage–specific pathways signify that the different pathways are controlled by the tumor cells, depending on the priority of specific tumor stages.

To elucidate the transcriptional circuits governing the correlation-enhancing and -repressing genes in the tumor, we constructed the TF–target gene network. Interestingly, the network analysis showed that a single TF is likely to possess the capacity to target both correlation-enhancing and -repressing genes. For instance, STAT1 may exert positive transcriptional control over the correlation-enhancing genes associated with metabolic reprogramming, PPP, WNT signaling, and chaperonin-mediated protein folding (Figure 6A). On the other hand, correlation-repressing genes linked to oxidative phosphorylation and vesicle-mediated transport are also likely to be transcriptionally negatively controlled by STAT1 (Figure 6A). According to TRRUST (transcriptional regulatory relationships unraveled by sentence-based text mining) (Han et al., 2018) and Uniprot (The UniProt, 2017), STAT1 can have both positive and negative effect on the gene expression. Previously, it has been proposed that STAT1 is involved in the regulation of the Warburg effect in the tumor cells by activating a switch from oxidative phosphorylation to anaerobic glycolysis (Pitroda et al., 2009). Moreover, the modulatory role of STAT1 on oxidative phosphorylation has been demonstrated by using STAT1^−/−^ mice (Sisler et al., 2015). Similar to STAT1, other TFs—STAT3, PML, KDM1A, and RELA—were found to have both positive and negative impacts on the regulation of transcription of correlation-enhancing and -repressing genes, respectively (Figure 6 and Supplementary Figure 6). These TFs were also annotated as activators and repressors of gene expression by TRRUST and Uniport. Diverse factors alone or in combination can be responsible for the differential activity of these TFs. For instance, transcriptional activation of STAT1 may rely upon the recruitment of coactivators and interaction with the core transcriptional machinery (Ramana et al., 2000). On the contrary, STAT1 transcriptional suppression may involve the modification of coactivators within the transcriptional complexes (Ramana et al., 2000). Moreover, modification patterns of STATs, such as unphosphorylated STATs control gene expression by novel mechanisms that are different from those used by phosphorylated STAT dimers (Yang and Stark, 2008). These findings underscore the possible roles of coactivator/repressor, post-translational modifications, and TF-binding motifs in the promoter of target genes in determining the activating or repressing function of TFs.

In summary, the current study illustrates the significant impact of the mRNA and protein stability on their subsequent correlation and showed the evolutionarily conserved hierarchical patterns of the stability-dependent correlation across diverse tissues and mammalian species. We further revealed a unique utility of the mRNA-to-protein correlation in the elucidation of the pathways likely under transcriptional control in tumor tissues. It has been proposed that transcriptional regulatory circuits governed by the master regulatory molecules are important for cancer cells to survive (Califano and Alvarez, 2017). By identifying the deviating mRNA-to-protein correlations, we were able to uncover the transcriptional networks governing the key pathways and metabolic reprogramming in tumor tissues in the early and late stages. However, further studies based on cancer cell-line models and clinical samples are required to gain a mechanistic understanding of how these identified transcriptional networks act in concert to drive tumor evolution.

## Data Availability Statement

The data that support the findings of this study are available in publicly available data repositories or published articles. The name of the data repositories along with their web addresses (URLs), accession numbers are given in the following “Web Resources” section and Supplementary Table 1. No data from human subjects other than from public repositories and published articles were used in this article.

## Author Contributions

GA, SC, TD, and MB conceptualized the project, wrote, corrected, and edited the paper. GA, SC, and TD performed the data retrieval, normalization, and downstream bioinformatics analysis of the data. All authors contributed to the article and approved the submitted version.

## WEB RESOURCES

All the R-scripts and source files used for generating the Figures are deposited in GitHub (https://github.com/gandrigit/Papers/tree/master/mRNA_Protein_stability).

Stability profile dataset: (PMID: 21593866).

Bioproject: https://www.ncbi.nlm.nih.gov/bioproject/?term=PRJNA80021

RNA-seq data: https://trace.ncbi.nlm.nih.gov/Traces/sra/?run=SRR149237

Proteomics data: https://www.nature.com/articles/nature10098#Sec10

RFP in mouse dendritic and human HEK cells: PMID: 25745177 and PMID: 26657557.

Bioproject (mouse): https://www.ncbi.nlm.nih.gov/bioproject/?term=PRJNA256211

Bioproject (HEK cells): https://www.ncbi.nlm.nih.gov/bioproject/?term=PRJNA296059

RNA-seq data (mouse): https://www.ncbi.nlm.nih.gov/geo/query/acc.cgi?acc=GSE59793

RNA-seq data (HEK cells): https://www.ncbi.nlm.nih.gov/geo/query/acc.cgi?acc=GSE73136

Proteomics data (mouse): ftp://MSV000078994:a@massive.ucsd.edu.

Proteomics data (HEK cells): https://www.ebi.ac.uk/pride/archive/projects/PXD002389

Human tissue mRNA abundance (mRNA-to-protein correlation Dataset 1): GTEx - PMID: 23715323.

Sample annotation: https://gtexportal.org/home/samplesPage

RNA-seq data: https://gtexportal.org/home/datasets

Human tissue protein abundance (mRNA-to-protein correlation Dataset 1): HPM - PMID: 24870542.

Proteomics data: https://www.ebi.ac.uk/pride/archive/projects/PXD000561

Human tissue mRNA and protein abundance (mRNA-to-protein correlation dataset 2): PMID: 24870543.

Sample annotation: https://www.proteomicsdb.org/proteomicsdb/#overview

RNA-seq data: https://www.nature.com/articles/nature13319#Sec10

Proteomics data: https://www.ebi.ac.uk/pride/archive/projects/PXD000865

Human tissue mRNA and protein abundance (mRNA-to-protein correlation dataset 3): PMID: 30777892

Sample annotation: www.uppsalabiobank.uu.se/en/

RNA-seq data: http://www.ebi.ac.uk/arrayexpress/experiments/E-MTAB-2836/

Proteomics data: https://www.ebi.ac.uk/pride/archive/projects/PXD010154

mRNA and protein abundance in human cell lines: PMID: 22278370.

RNA-seq and proteomics data: https://www.mcponline.org/content/suppl/2012/01/25/M111.014050.DC1

mRNA and protein abundance in colon tumor: PMID: 25043054.

Sample annotation: https://cptac-data-portal.georgetown.edu/study-summary/S022

RNA-seq data:

Proteomics data: https://www.ebi.ac.uk/pride/archive/projects/PXD002089

Protein abundances in non-tumor colon: CPTAC.

Sample annotation: https://cptac-data-portal.georgetown.edu/study-summary/S019

Proteomics data: https://cptc-xfer.uis.georgetown.edu/publicData/Phase_II_Data/Normal_Colon_Epithelium/.

## Conflict of Interest

The authors declare that the research was conducted in the absence of any commercial or financial relationships that could be construed as a potential conflict of interest.
