# Supplementary material for: Alteration of Proteotranscriptomic Landscape Reveals the Transcriptional Regulatory Circuits Controlling Key-Signaling Pathways and Metabolic Reprogramming During Tumor Evolution
Source: Front Cell Dev Biol. 2020 Dec 15;8:586479. doi: 10.3389/fcell.2020.586479 (PMC7769845; doi:10.3389/fcell.2020.586479)
Supplement: Supplementary file 11 [file Data_Sheet_1.pdf]

Supplementary Material

1 Supplementary Figures

Supplementary Figure 1

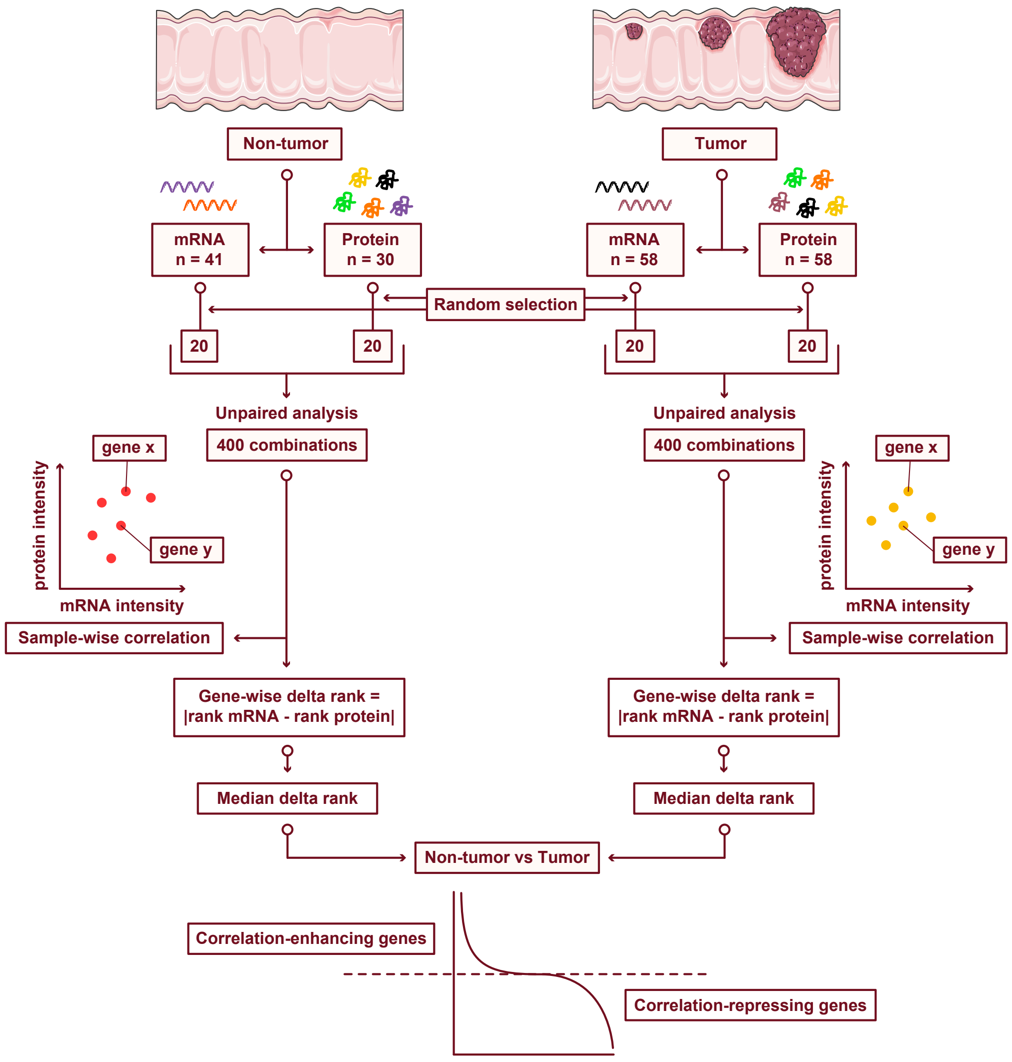

Supplementary Figure S1: Schematic flow diagram representing the randomization strategy of unpaired correlation analysis comparing tumor and non-tumor tissues.

Supplementary Figure 2

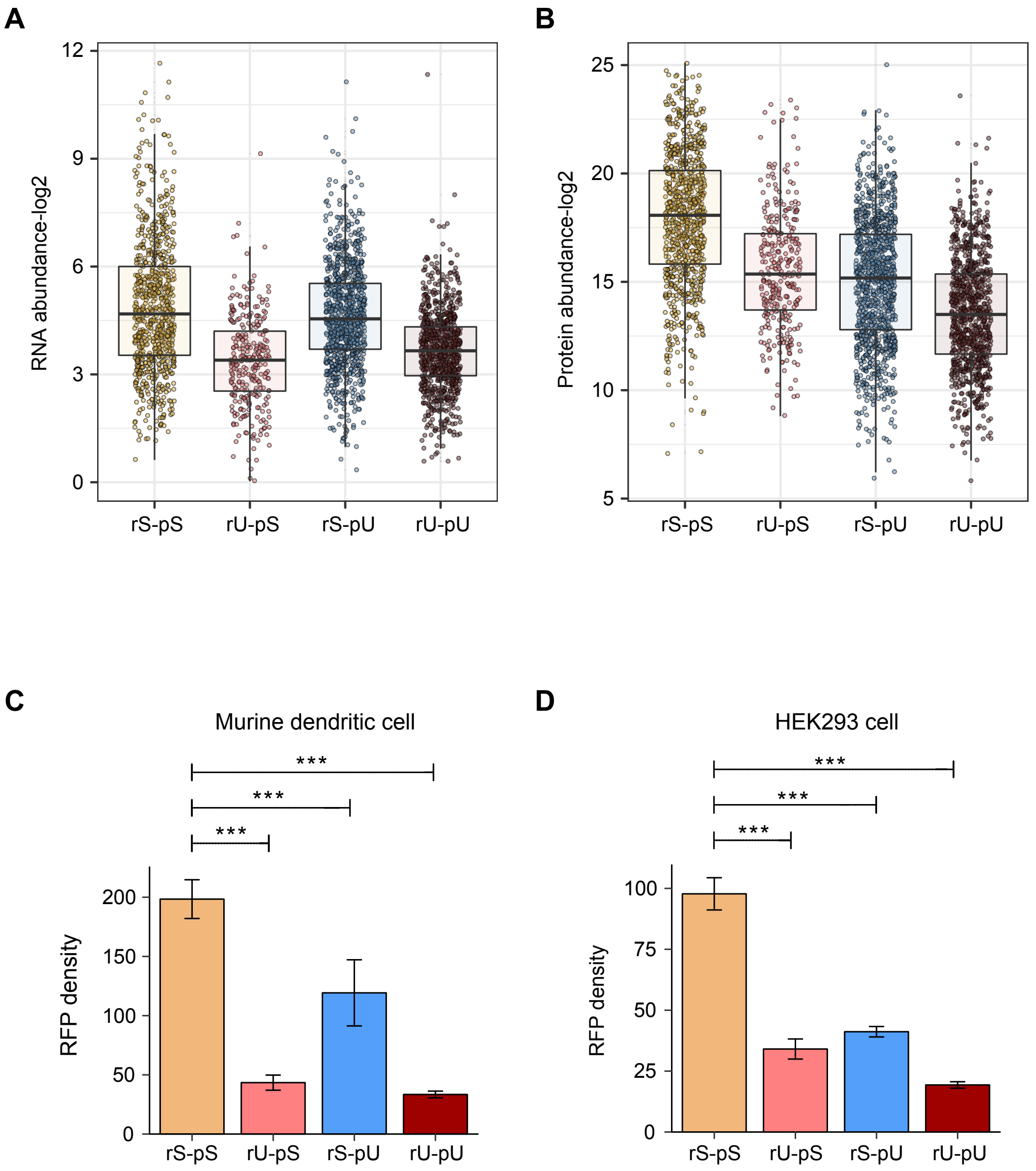

Supplementary Figure S2: Distribution of mRNA and protein abundances (A, B), and ribosomal footprinting (C, D) across different stability group. Differential mRNA and protein abundance according to the stability group. Boxplot showing the distribution of the mRNA (A) and protein (B) across the 4 stability groups. The median and inter-quartile range of mRNA-to-protein correlation (Spearman's correlation) in each stability groups is shown in the box plot. Barplots showing the average ribosomal footprinting (RFP) density values in different stability groups in murine dendritic cells (C) and Human HEK-293 cells (D). Error bars represent the standard error of the mean. The significance of the two-sided Wilcoxon test is indicated as follows: *** FDR < 0.001.

Supplementary Figure 3

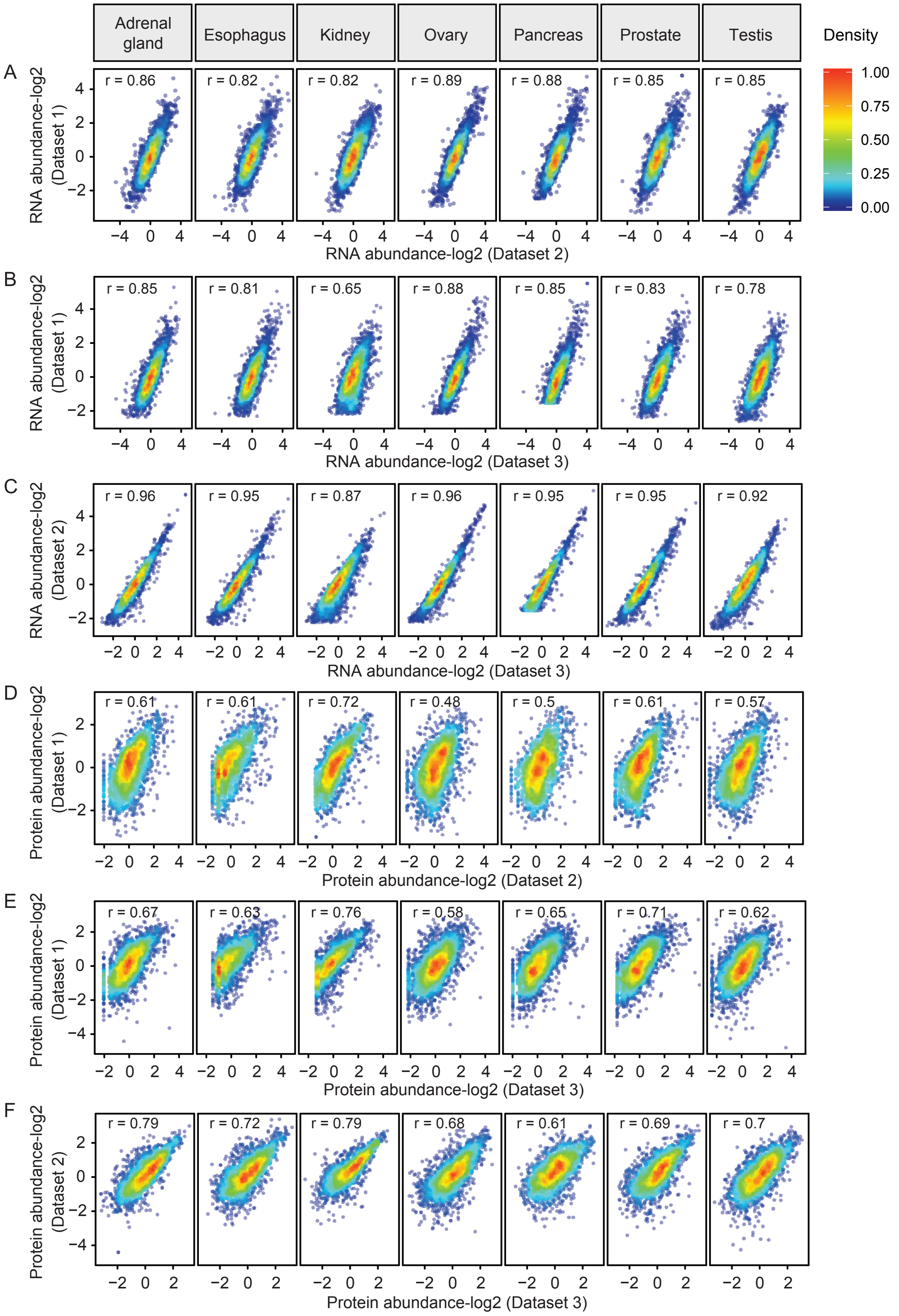

Supplementary Figure S3: mRNA-to-mRNA and protein-to-protein crossmatching across different datasets. mRNA-to-mRNA (A, B, C) and protein-to-protein (D, E, F) correlation scatterplots across Datasets-1, -2 and -3. (A) mRNA Dataset-1 vs. Dataset-2, (B) mRNA Dataset-1 vs. Dataset-3, (C) mRNA Dataset-2 vs. Dataset-3, (D) protein Dataset-1 vs. Dataset-2, (E) Dataset-1 vs. Dataset-3, (F) Dataset-2 vs. Dataset-3. mRNA and protein abundances were sample-wise scaled using z-score to make their range uniform. R indicates Spearman's correlation significant. Color code represents the relative density.

Supplementary Figure 4

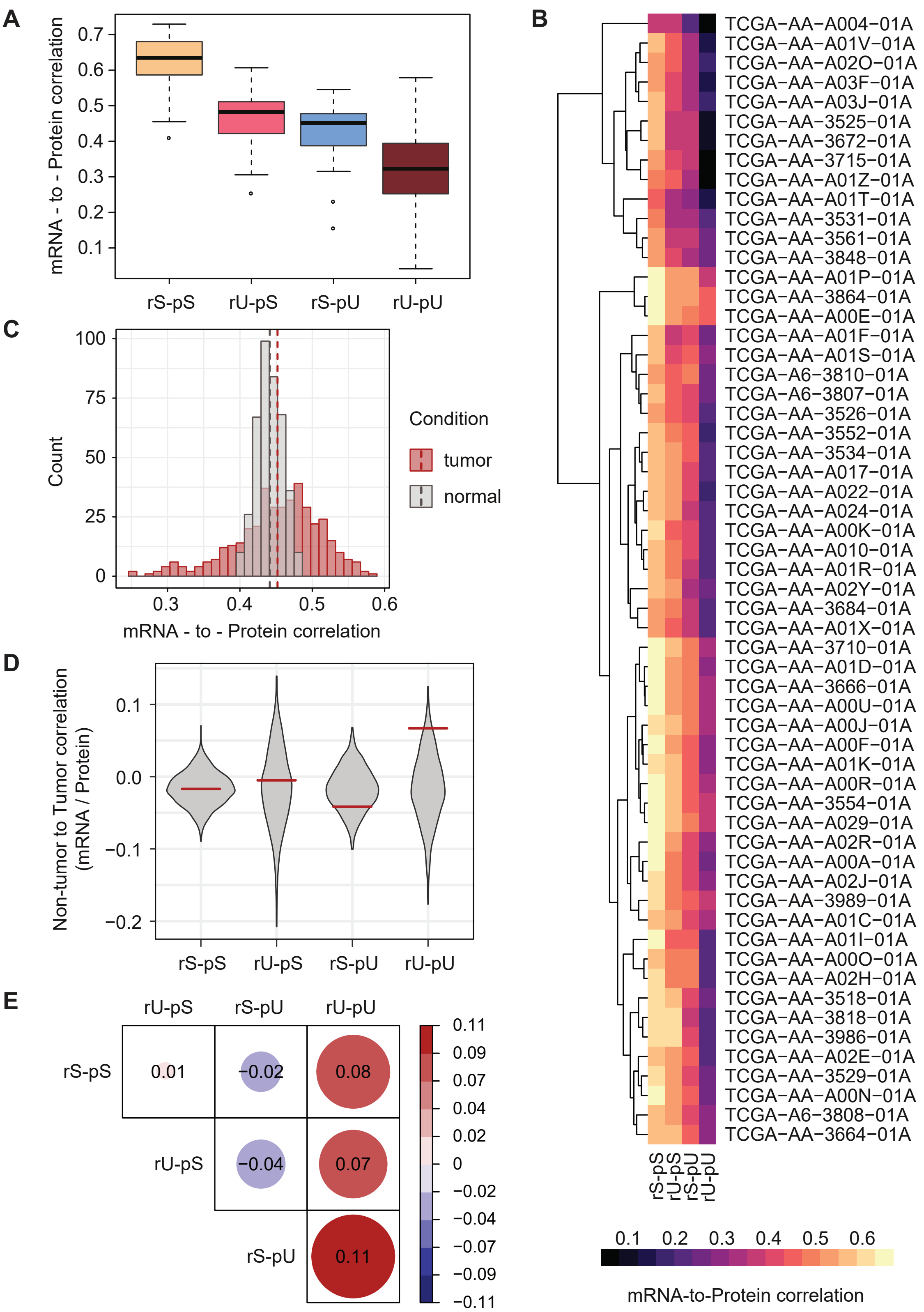

Supplementary Figure S4: mRNA-to-protein correlation in TCGA COAD (A) Boxplot displaying the differential mRNA-to-Protein correlation across the four stability groups in the 58 COAD samples. Boxplot represents the median and inter-quartile range of mRNA-to-protein correlation (Spearman's correlation) in each stability group (B) Heatmap showing the individual mRNA-to-Protein correlation value in the 58 COAD samples. Rows were clustered using hierarchical clustering on Euclidean distance. (C) Histogram showing the distribution of the overall mRNA-to-Protein Spearman's correlation in tumors compared to non-tumor. Average correlation in each condition is represented by a dashed line. (D) Heatmap showing the difference of mRNA-to-protein correlation between stability groups in rows and columns. Color code represents the difference in correlation. The circle size is proportional to the absolute difference of correlation. (E) Violin plot showing the visual representation of the expected difference of correlation between non-tumor and tumor condition (grey violin plot) against the observed difference (red line).

Supplementary Figure 5

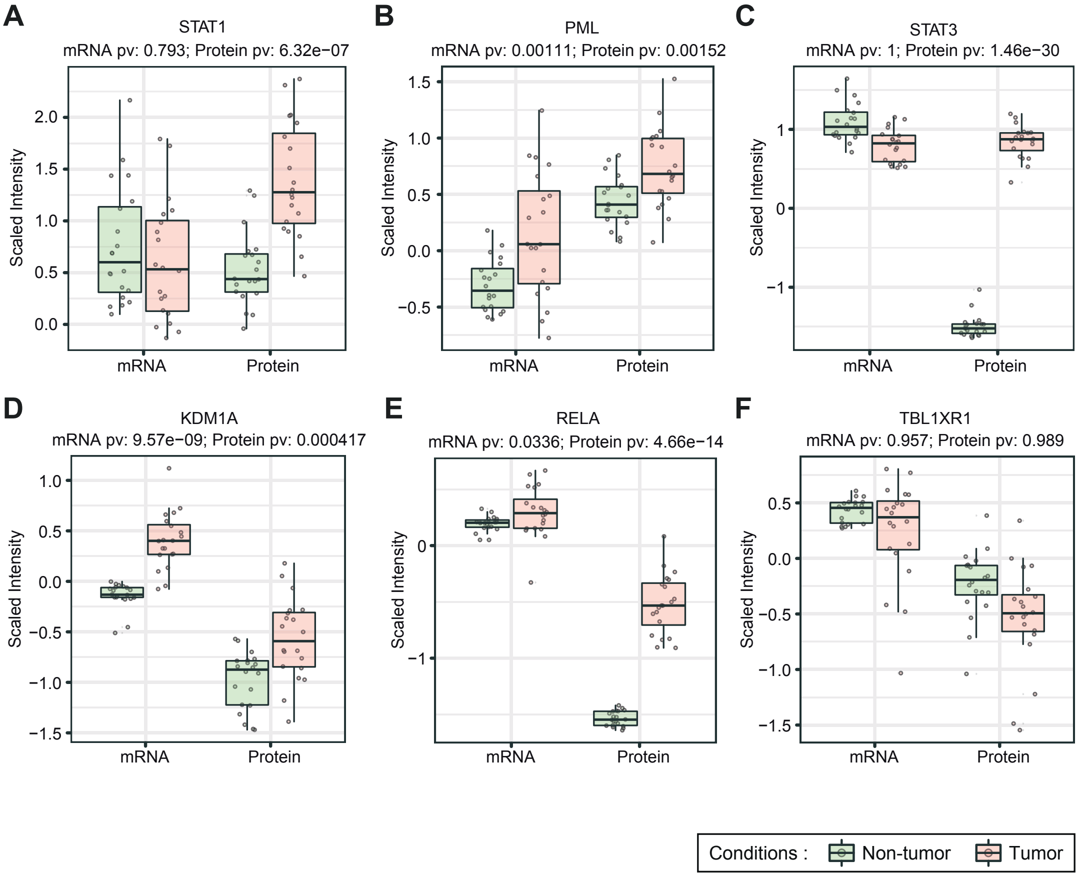

Supplementary Figure S5: Differential mRNA and protein abundances of transcription factors (TFs) between tumor and non-tumor tissues. Boxplots showing the median and inter-quartile range of mRNA and protein abundances of STAT1 (A), PML (B), STAT3 (C), KDM1A (D), RELA (E), and TBL1XR1 (F) between tumor and non-tumor tissues are shown. Statistical significance obtained by a t-test is shown on top, for both mRNA and protein, where a significant p-value indicates a higher mRNA or protein abundances in tumor compared to non-tumor tissues. Color code indicates the tumor (red) and non-tumor (green) samples. mRNA and protein abundances were scaled in a sample-wise manner by using z-score.

Supplementary Figure 6

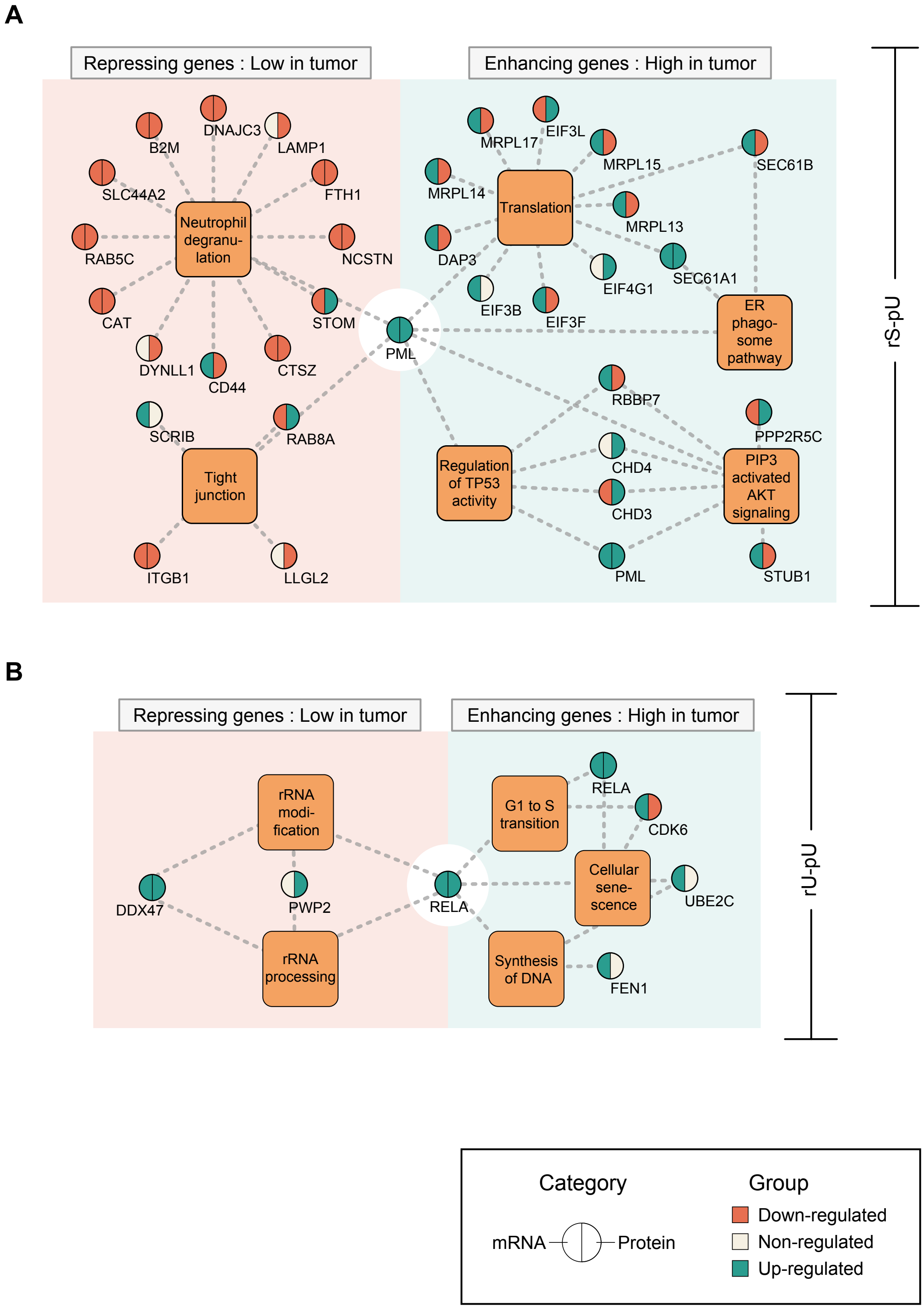

Supplementary Figure S6: Transcription factor (TF) - target gene network. Transcriptional regulatory networks designed by the TF-target gene association are shown. The TF-target networks describing the transcriptional control of correlation-enhancers and -repressors gene are depicted for PML network for rS-pU genes (A), and RELA network for rU-pU (B) genes. TFs to target genes relationship was established based on Encode data. TFs are connected to their know targets through the enriched processes in tumors compared to non-tumor tissue from Figure 5. Color code indicates the regulatory pattern of mRNAs/proteins in tumor tissues (Green indicates upregulation in tumor whereas red indicates downregulation in the tumor) as defined by the p-value (P-value bellow 0.05 was set as significant and considered as regulated). The left and right parts of the nodes delineate mRNA and protein regulations.

Supplementary Figure 7

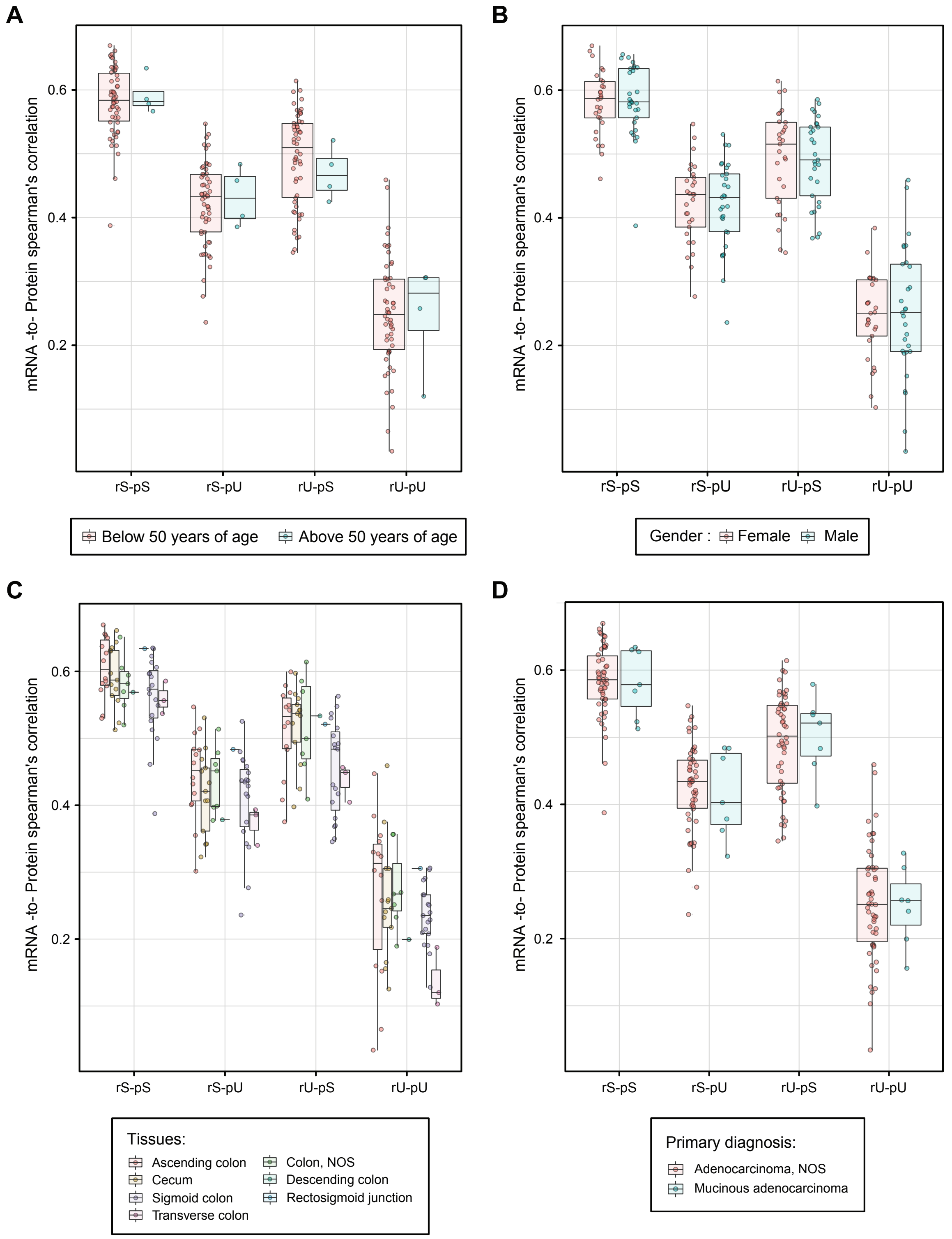

Supplementary Figure S7: Effect of age (A), gender (B), tumor tissue location (C) and primary diagnosis (D) on mRNA-to-protein correlation. The boxplots are showing the median and inter-quartile range of mRNA-to-protein correlation (Spearman's correlation) in each stability groups. Each dot represents a different patient where we have matched mRNA and protein sample. The list of these patients is available in Supplementary Table S7. To analyze the effect of age, patients were divided into two categories: below 50 and above 50 years of age.

Supplementary Figure 8

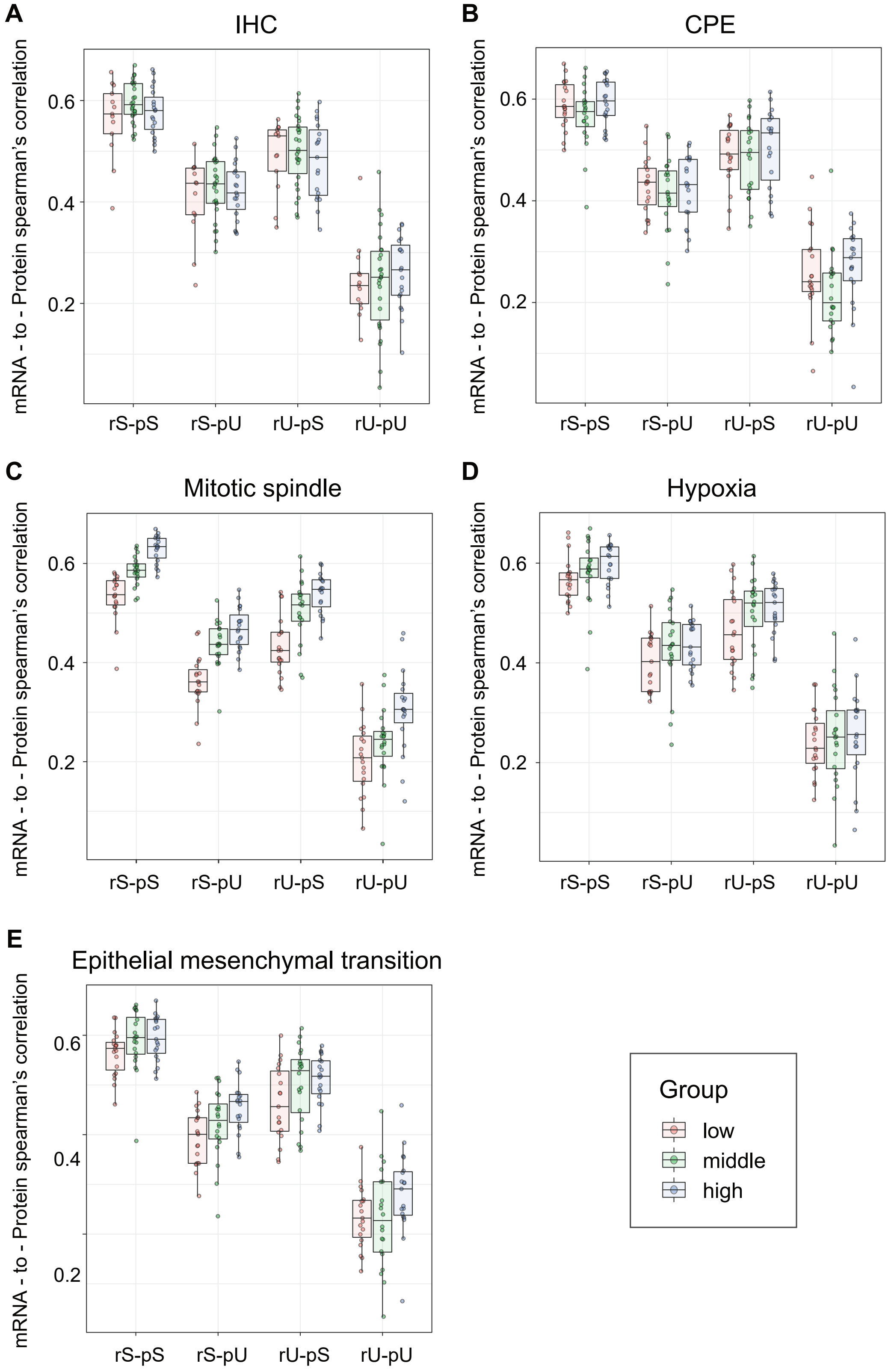

Supplementary Figure S8: Effect of tumor purity (A and B) and molecular features (D-E) on mRNA-to-protein correlation. The boxplots representing the median and inter-quartile range of mRNA-to-protein correlation (Spearman's correlation) in each stability group are shown. Each dot represents a different patient where matched mRNA and protein samples are available. To analyze the effect of tumor purity, two parameters – immunohistochemistry (ICH) (A) and Consensus Purity Estimation (CPE) (B) were used. Patients were divided into three groups (high, intermediate and low) based on 3-quantiles using ICH and CPE scores. For molecular features: Mitotic Spindle (C), Hypoxia (D) and Epithelial to Mesenchymal Transition (EMT) (E). Normalized Enrichment Score (NES) was classified into three groups (high, intermediate and low) based on 3-quantiles.

Supplementary Figure 9

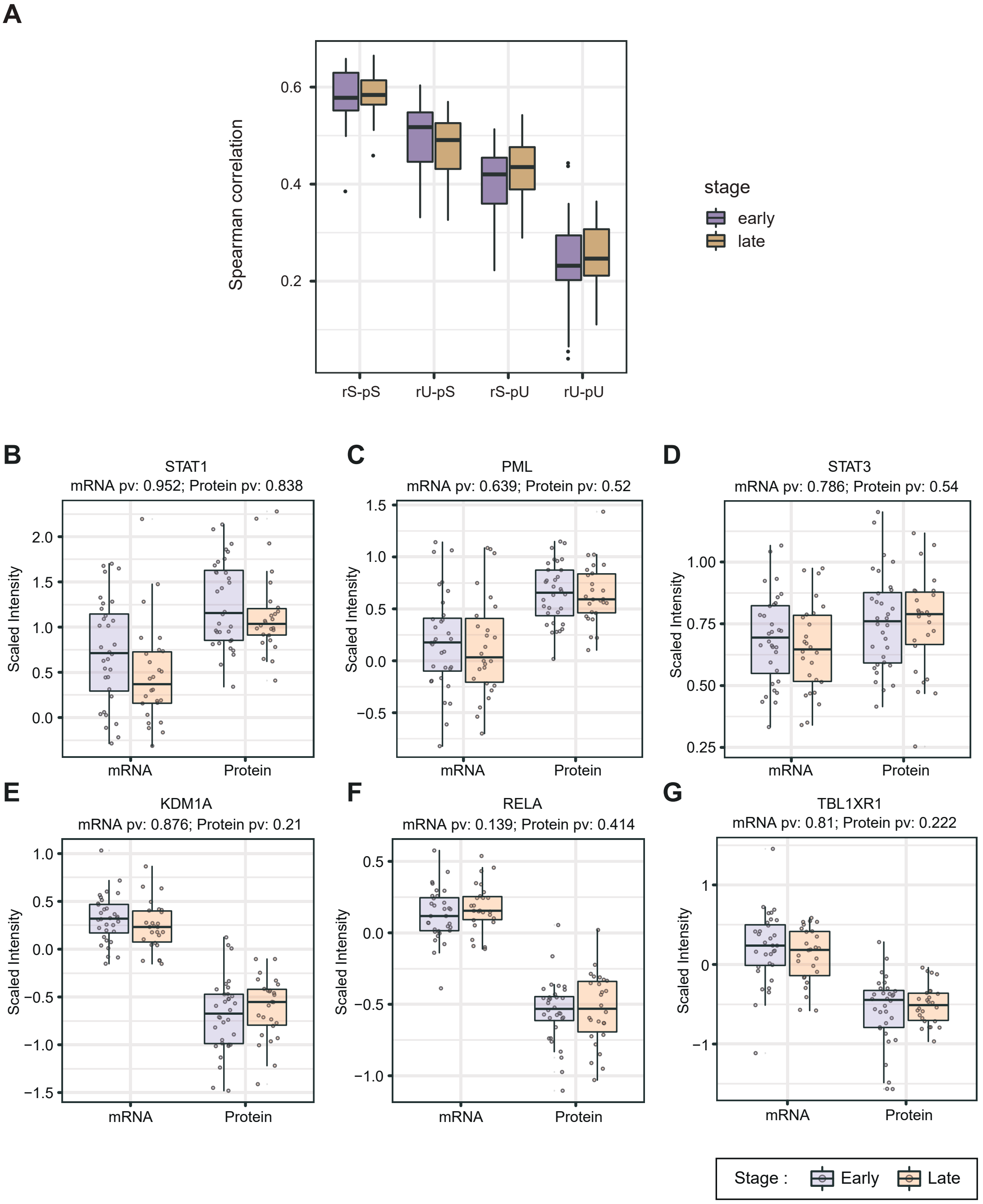

Supplementary Figure S9: stability-dependent mRNA-to-Protein correlation in COAD early and late stages. (A) Distribution of the mRNA-to-protein correlation in early and late COAD stages was obtained in paired analysis across different stability groups. The correlation values are visualized on a boxplot representing the median and inter-quartile range of mRNA-to-protein. Color code indicates the early and late stages. mRNA and protein intensity of STAT1 (B), PML (C), STAT3 (D), KDM1A (E), RELA (F), TBL1XR1 (G) in the early and late stages of COAD tissues. Statistical significance from the t-test is written on top, for both mRNA and protein, where a significant p-value indicates higher expression in late compared to early stage. Color code indicates the early and late stages. mRNA and protein abundances were sample-wise scaled using z-score.

2 Supplementary Tables

Supplementary Table S1: Sample sources, transcriptomics and proteomics data repositories, inclusion/exclusion criteria, tumor and non-tumor sample histology, clinical and demographic features of colon cancer patients.

Supplementary Table S2: mRNA and protein half-lives defining the stability groups, based on Schwanhausser et al (Schwanhausser et al., 2011) and human orthologous genes.

Supplementary Table S3: Stability-dependent Ribosomal Footprint density in murine dendritic cell (Jovanovic et al., 2015) and HEK293 cell (Calviello et al., 2016).

Supplementary Table S4: Stability-dependent mRNA and protein intensities from different human tissues (Consortium, 2013; Kim et al., 2014; Wilhelm et al., 2014; Wang et al., 2019).

Supplementary Table S5: Stability-dependent mRNA and protein intensity in cell-lines (Geiger et al., 2012).

Supplementary Table S6: stability-dependent mRNA and protein intensity in the colon tumor (COAD) and non-tumor colon tissues based on TCGA (Zhang et al., 2014) and CPTAC (Edwards et al., 2015).

Supplementary Table S7: Classification of COAD patients according to tumor stages, age groups, gender, tissue of origin and histologic subtypes.

Supplementary Table S8: Comparative data distribution analysis.

Supplementary Table S9: Summary of bootstrap analysis demonstrating the significance of the mRNA-to-Protein correlation in each stability group in Dataset-1, -2 and -3.

Supplementary Table S10: Bootstrap analysis of tissue vs. cell line.
